# GSAR: Bioconductor package for Gene Set analysis in R

**DOI:** 10.1186/s12859-017-1482-6

**Published:** 2017-01-24

**Authors:** Yasir Rahmatallah, Boris Zybailov, Frank Emmert-Streib, Galina Glazko

**Affiliations:** 1 Department of Biomedical Informatics, University of Arkansas for Medical Sciences, Little Rock, AR 72205 USA; 2 Department of Biochemistry and Molecular Biology, University of Arkansas for Medical Sciences, Little Rock, AR 72205 USA; 3 Computational Medicine and Statistical Learning Laboratory, Tampere University of Technology, Korkeakoulunkatu 1, Tampere, FI-33720 Finland

**Keywords:** Gene set analysis, Non-parametric, Pathways, Kolmogorov-Smirnov, Wald Wolfowitz, Minimum spanning tree

## Abstract

**Background:**

Gene set analysis (in a form of functionally related genes or pathways) has become the method of choice for analyzing omics data in general and gene expression data in particular. There are many statistical methods that either summarize gene-level statistics for a gene set or apply a multivariate statistic that accounts for intergene correlations. Most available methods detect complex departures from the null hypothesis but lack the ability to identify the specific alternative hypothesis that rejects the null.

**Results:**

GSAR (Gene Set Analysis in R) is an open-source R/Bioconductor software package for gene set analysis (GSA). It implements self-contained multivariate non-parametric statistical methods testing a complex null hypothesis against specific alternatives, such as differences in mean (shift), variance (scale), or net correlation structure. The package also provides a graphical visualization tool, based on the union of two minimum spanning trees, for correlation networks to examine the change in the correlation structures of a gene set between two conditions and highlight influential genes (hubs).

**Conclusions:**

Package GSAR provides a set of multivariate non-parametric statistical methods that test a complex null hypothesis against specific alternatives. The methods in package GSAR are applicable to any type of omics data that can be represented in a matrix format. The package, with detailed instructions and examples, is freely available under the GPL (> = 2) license from the Bioconductor web site.

**Electronic supplementary material:**

The online version of this article (doi:10.1186/s12859-017-1482-6) contains supplementary material, which is available to authorized users.

## Background

The idea of considering functional units (e.g., molecular pathways) instead of individual components (e.g., genes) in studying omics data was first employed by Mootha and colleagues [1], in analyzing microarray gene expression data of diabetic subjects against healthy controls. While analysis of individual gene expressions did not detect any significant changes, the pathway level approach named Gene Set Enrichment Analysis (GSEA) indicated that the group of genes involved in oxidative phosphorylation was overall under-expressed in diabetics although individual genes were on average only 20% under-expressed in diabetics [1]. Since that time, many methodologies for finding differentially expressed gene sets have been suggested and are collectively named Gene Set Analysis (GSA) approaches [2, 3]. The benefits of pathways analysis can be summarized as follows. First, pathway level analysis incorporates accumulated biological knowledge into the results and conveys more explanatory power than a long list of seemingly unrelated differentially expressed genes [3]. Second, pathway analysis accounts for intergene correlations and facilitates the detection of small or moderate changes in genes expression that could be overlooked by univariate tests. Third, by arranging genes in pathways (gene sets) the number of simultaneously tested hypotheses is reduced, increasing the detection power after applying correction for multiple testing. These benefits made pathway analysis the method of choice in analyzing omics data in general and gene expression data in particular.

GSA approaches can be either *competitive* or *self-contained*. Competitive approaches compare a gene set in two conditions against its complement that consists of all the genes in the dataset excluding the genes in the set itself, and self-contained approaches test if a gene set is differentially expressed between two experimental conditions. Some competitive approaches can be influenced by data filtering and the size of the dataset [4] while others can be influenced by the proportion of up-regulated and down-regulated genes in a gene set between two experimental conditions [5]. While competitive approaches test the same alternative hypothesis against null hypothesis, self-contained approaches provide the flexibility of testing different alternative hypotheses that may correspond to different biological phenomena thus increasing the biological interpretability of experimental results. We distinguish the three major alternative hypotheses that can be tested by GSA approaches: (1) differential expression (DE); (2) differential variability (DV); and (3) differential co-expression or correlation (DC) of gene sets between two conditions. Package GSAR (Gene Set Analysis in R) provides a set of self-contained non-parametric multivariate GSA methods that test each of these three different hypotheses.

The majority of GSA methods were developed to identify gene sets with differences in mean gene expressions between two conditions for microarray or RNA-seq data. Some DE tests were true multivariate methods (e.g., ROAST [6]) while others aggregate the outcome of gene-level univariate tests (e.g., SAM-GS [7]). *N*-statistic [8] tests more general alternative hypothesis whether two multivariate distributions are different. Detailed discussion and comparative power analysis for selected methods is presented in [5, 9]. Package GSAR implements one multivariate method (WWtest) to identify differences in distributions between two conditions and two non-parametric multivariate methods to identify differences in mean expressions (KStest and MDtest) using sample ranking based on the minimum spanning trees (MSTs) [10]. The analysis of DV for individual genes identifies genes with significant changes in expression variance between two conditions [11–15]. The DV analysis frequently complements or provides more relevant explanations for biological phenomena than simple difference in mean expressions. For example, a theoretical model for evolutionary fitness suggested that increased gene expression variability is a defining characteristic of cancer [16]. This suggestion was further supported by the observation of increased variability in DNA methylation of specific genes across five different cancer types [17]. Moreover, some genes were found to show consistently hyper-variability in tumors of different origins as compared to normal samples [18] and such genes can serve as a robust molecular signature for multiple cancer types [18, 19]. Although software packages for univariate methods testing DV are available, to the best of our knowledge multivariate methods for gene set DV analysis are non-existing. Package GSAR implements two non-parametric DV approaches: (1) an approach that uses the aggregation of *P*-values from univariate *F*-tests as a test statistic and sample permutations to estimate the null distribution of the statistic; and (2) a multivariate approach that tests the hypothesis of differential *p*-dimensional sample variability between two conditions using MST-based sample ranking with two different statistics [10].

In addition to tests of differential mean and variance, GSAR implements the Gene Set Net Correlation Analysis (GSNCA) method that tests a multivariate null hypothesis that there is no change in the net correlation structure of a gene set between two conditions [20]. It examines how the regulatory relationships and concordance between gene expressions vary between phenotypes. GSA approaches for identifying the differential gene set co-expression (correlation) have been also described in literature. For example, Gene Sets Co-expression Analysis (GSCA) aggregates the pairwise correlation differences between two conditions [21], while other methods such as the differentially Co-expressed gene Sets (dCoxS) aggregates differences in relative entropy [22]. Other approaches for the differential co-expression analysis of gene sets account for changes in aggregated measures of pairwise correlations [23, 24]. Yet another category of methods such as the Co-expression Graph Analysis (CoGA) identifies co-expressed gene sets by testing the equality of spectral distributions [25]. For each experimental condition CoGA constructs a full network from pairwise correlations and compares the structural properties of the two networks by applying Jensen-Shannon divergence as a distance measure between the graph spectrum distributions [25, 26]. Package GSAR implements the GSNCA method [20] that assesses multivariate changes in the gene co-expression network between two conditions but does not require network inference step. Net correlation changes are estimated by introducing for each gene a weight factor that characterizes its cross-correlations in the co-expression networks. Weight vectors in both conditions are found as eigenvectors of correlation matrices with zero diagonal elements. GSNCA tests the hypothesis that for a gene set there is no difference in the gene weight vectors between two conditions [20]. Package GSAR pairs the GSNCA method with a graphical visualization that uses MSTs of the correlation networks to examine the change in the correlation structures of a gene set between two conditions and highlight the most influential (hub) genes. This visualization facilitates interpretation of changes in a gene set.

In what follows we provide detailed description of the methods implemented in package GSAR, which is available from the Bioconductor project [27], and illustrate its potential applications. The similarity and differences between GSAR and other methods, implementing GSA approaches (mostly available as Bioconductor packages) are summarized in Additional file 1: Table S1.

## Implementation

**Fig. 1:**
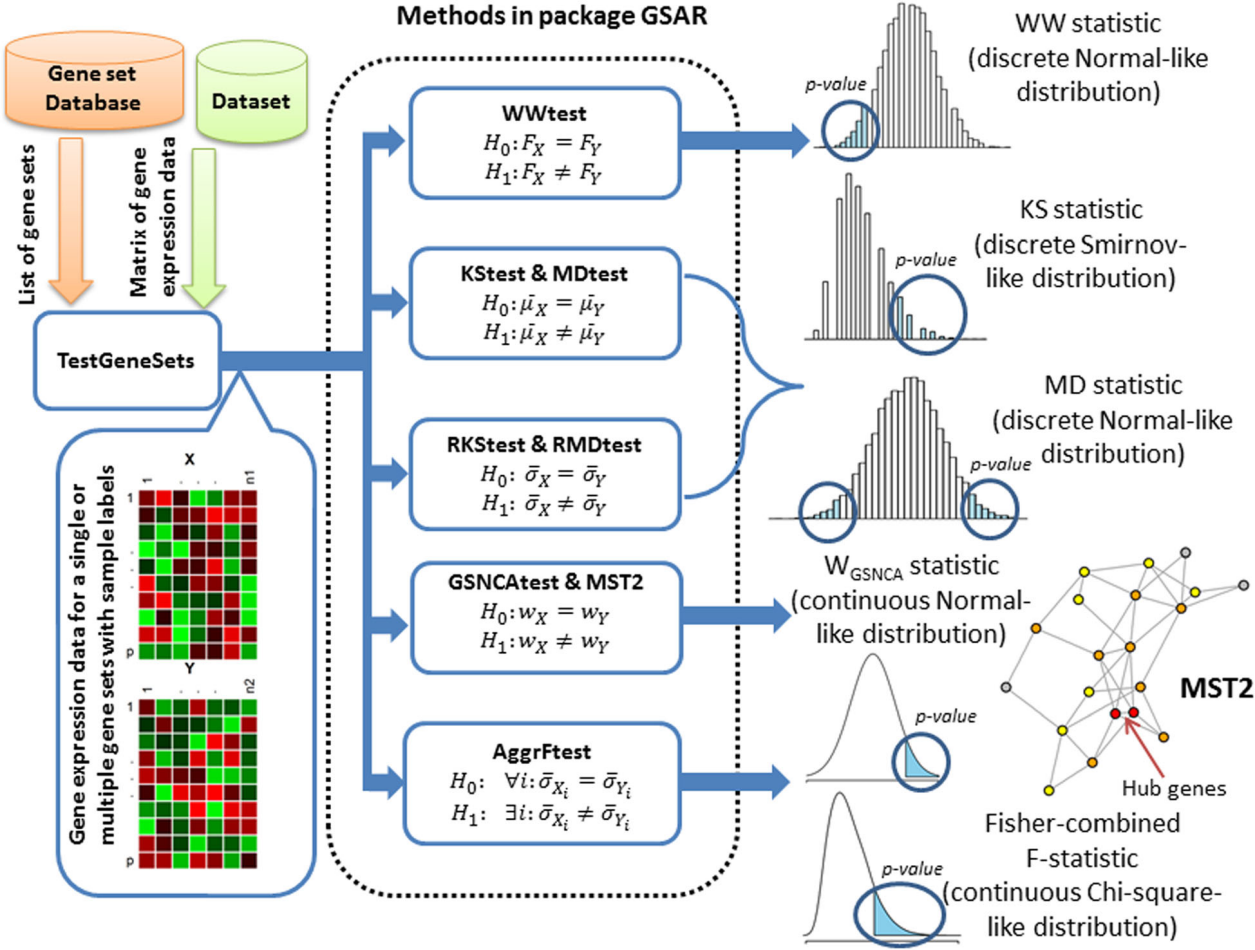
GSAR package outline. The inputs for the statistical tests can be (1) the matrix of gene expression for a single gene set in the form of normalized microarray or RNA-seq data and a vector of labels indicating to which condition each sample belongs; or (2) the matrix of gene expression for all genes, a vector of labels indicating to which condition each sample belongs, and a list of gene sets. Each test returns *P*-value and, optionally, the test statistic of observed data, test statistic for all permutations, and other optional outputs. Some functions produce graph plots

Package GSAR has been implemented in R [27] and employs the igraph class in package igraph [28] to handle and manipulate graph objects. Some of its implemented methods were developed and tested in [10, 20] and others are novel. Some of the methods in package GSAR are based on the multivariate generalizations of the Wald-Wolfowitz (WW) and Kolmogorov-Smirnov (KS) tests presented in [29]. All the statistical tests implemented are non-parametric in a sense that significance is estimated using sample permutations. A schematic overview of package GSAR is shown in Fig. 1. The minimum required input to any GSAR function that tests a single gene set consists of: (1) a gene expression matrix with *p* genes (rows) and *N* = *n*_1_ + *n*_2_ samples (columns); and (2) sample labels indicating to which phenotype each sample belongs in a form of integer numbers 1 and 2. All statistical methods return results as a list including *P*-values, test statistics for observed samples, and test statistics for permuted samples.

### Hypothesis testing

**Fig. 2:**
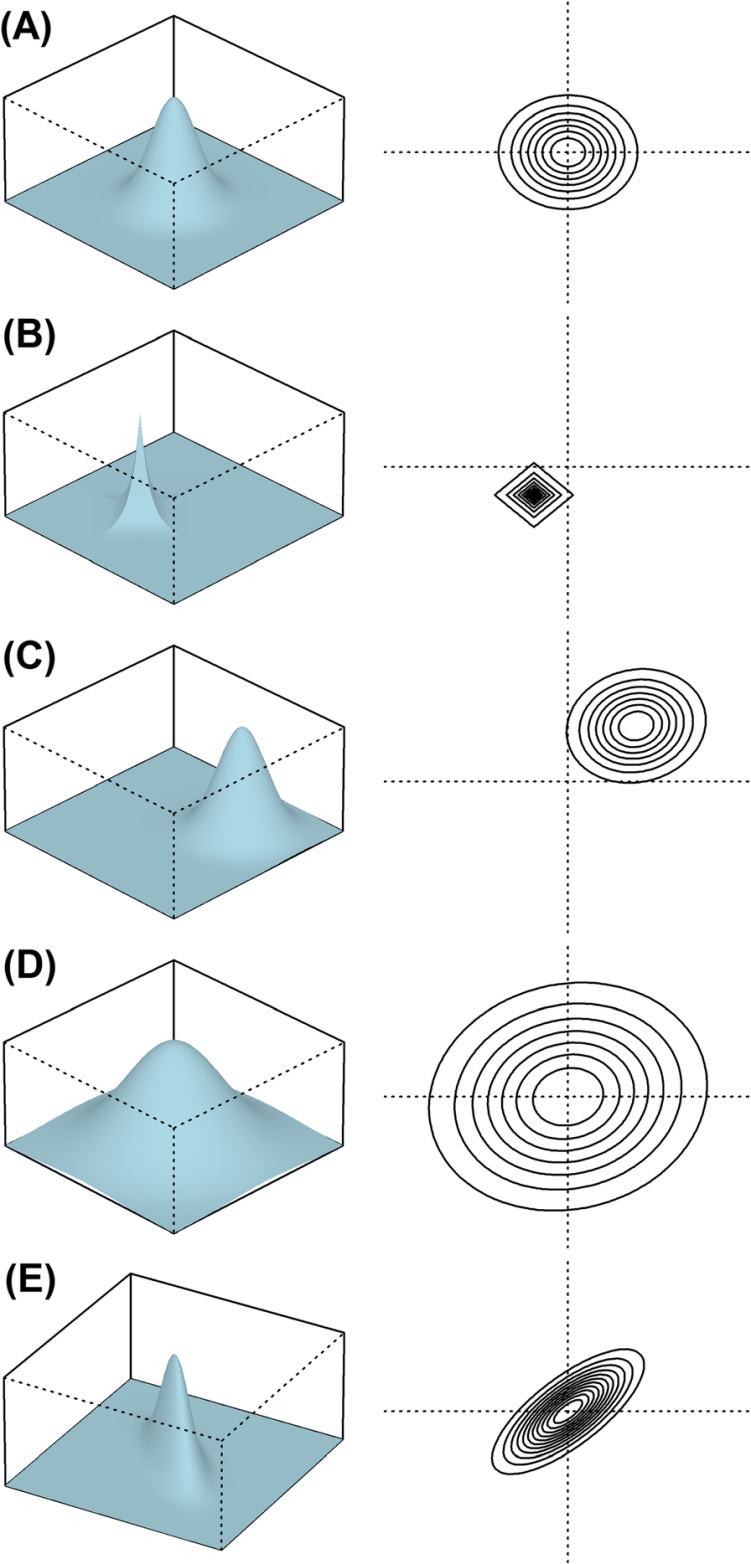
An example of bivariate normal distribution illustrating specific alternative hypotheses for a hypothetical gene set of size *p* = 2. **a** The standard bivariate normal distribution; **b** differential distribution as compared to panel A; **c** differential mean (shift) as compared to panel A; **d** differential variance (scale) as compared to panel A; **e** differential correlation between two components as compared to panel A. While the left-side panel shows three-dimensional density plots, the right-side panel shows the corresponding contour plots

Consider two different biological phenotypes (conditions), with *n*_1_ samples of measurements for the first and *n*_2_ samples of the same measurements for the second. Let $X={\left\{{x}_{ij}\right\}}_{px{n}_1}$ and $Y={\left\{{y}_{ij}\right\}}_{px{n}_2}$ represent the normalized measurements of *p* gene expressions of a gene set (pathway) in two phenotypes where sample *X*_*.j*_(*Y*_*.j*_) is the *j* ^th^ *p*-dimensional vector in one phenotype. Let *X*, *Y* be independent and identically distributed with the distribution functions *F*_*x*_, *F*_*y*_, *p*-dimensional mean vectors ${\overline{\mu}}_x$ and ${\overline{\mu}}_y$, and *p* × *p* positive-definite and symmetric covariance matrices *C*_*x*_ and *C*_*y*_. Statistical methods in package GSAR can test specific alternative hypotheses. Figure 2 illustrates these different alternative hypotheses using the simple example of a bivariate normal distribution which represent a gene set of size *p* = 2. The standard bivariate normal distribution shown in panel A of Fig. 2 is compared to the different alternatives shown in panels B, C, D, and E which show different distribution, mean (shift), variance (scale), and correlation as compared to panel A. Some alternative hypotheses are more specific than others. For example, different mean or variance implies different distribution; however the opposite is not necessarily true.

### Minimum spanning tree (MST)

The *p*-dimensional *N* samples from two phenotypes *X* and *Y* can be represented by an edge-weighted undirected graph *G*(*V*,*E*) with vertices *V* = {*v*_1_, ⋯, *v*_*N*_} corresponding to samples and edge weights estimated by the Euclidean distance between samples in the *R* ^*p*^ space. The minimum spanning tree (MST) of a graph *G*(*V*,*E*) is defined as the acyclic subset of edges *T*_1_ ⊆ *E* that is selected from the full set of *N*(*N*-1)/2 possible edges in the graph to connect all *N* vertices such that ${\sum}_{i,j\in {T}_1}d\left({v}_i,{v}_j\right)$ is minimal [29]. The distance between vertices *i* and *j* in the MST, *d*(*v*_*i*_, *v*_*j*_), correlates with their distance in *R* ^*p*^. This property allows the multivariate generalization of multiple univariate test statistics so they could be used for *p*-dimensional expression data of gene sets. The MST is built using a standard function from package igraph where the used algorithm is selected automatically. For weighted graphs, Prim’s algorithm is chosen.

### Required input

The required input for all the test functions in the package GSAR can be in two modes: (1) single gene set mode, where the matrix of gene expressions (or other gene-related measurements, e.g., protein abundances) for a single gene set and a numeric vector specifying the experimental group or condition for each sample are provided; (2) multiple gene sets mode, where the full matrix of gene expressions, a numeric vector specifying the experimental condition, and a list of gene sets (each item is a character vector of gene identifiers) are provided (function TestGeneSets).

### Data and examples

A processed version of the p53 dataset is included in the package for demonstration and can be loaded using data(p53DataSet). This dataset comprises 50 samples of the NCI-60 cell lines: 17 cell lines carrying wild type (WT) TP53 and 33 cell lines carrying mutated (MUT) TP53 [30]. Transcriptional profiles obtained from Affymetrix microarrays (platform hgu95av2) were downloaded from the Broad Institute's website. The processing steps are listed in the reference manual and the package vignette (see Additional file 2). The vignette also demonstrates the use of different tests in GSAR using the ALL (microarray) and Pickrell (RNA-seq) datasets, available respectively from the Bioconductor data packages ALL and tweeDEseqCountData.

## Results and discussion

This Section presents the statistical methods available in package GSAR that test different statistical hypotheses. Several examples based on simulated and real datasets illustrate methods application.

### Multivariate Wald-Wolfowitz test

In the multivariate WW test, the MST is constructed and all the edges connecting two vertices from different phenotypes are removed to split the MST into disjoint trees. The standardized number of remaining disjoint trees (*R*) is used as the test statistic [10, 29]

$$ W=\frac{R-E\left[R\right]}{\sqrt{var(R)}} $$

The null distribution is estimated by permuting sample labels and calculating *R* for a large number of times *M*. The null distribution is asymptotically normal and *H*_0_ is rejected for a small number of subtrees [29]. The significance (*P*-value) is calculated as

$$ P- valu{e}_{WW}=\frac{{\displaystyle {\sum}_{k=1}^M}\;I\left[{W}_k\le {W}_{obs}\right]+1}{M+1} $$

where *W*_*k*_ is the test statistic of permutation *k*, *W*_*obs*_ is the observed test statistic from the original data and *I*[.] is an indicator function. Function WWtest in package GSAR implements this method, testing the null hypothesis *H*_0_ : *F*_*X*_ = *F*_*Y*_ against the alternative *H*_1_ : *F*_*X*_ ≠ *F*_*Y*_, where *F*_*X*_ and *F*_*Y*_ are the distribution functions of *X* and *Y*, respectively. The following R command implements the method

WWtest(object, group, nperm = 1000, pvalue.only = TRUE)

where object is a numeric matrix of gene expression with columns and rows corresponding to samples and genes, respectively, group is a numeric vector (with values 1 and 2) indicating group associations for samples, nperm is the number of permutations used to estimate the null distribution, and *p*value.only is a logical parameter that indicates if returning the *P*-value only is desired. When *p*value.only = FALSE, the observed statistic, the vector of permuted statistics, and the *P*-value are returned in a list (see Additional file 2 for examples of real dataset analysis).

**Fig. 3:**
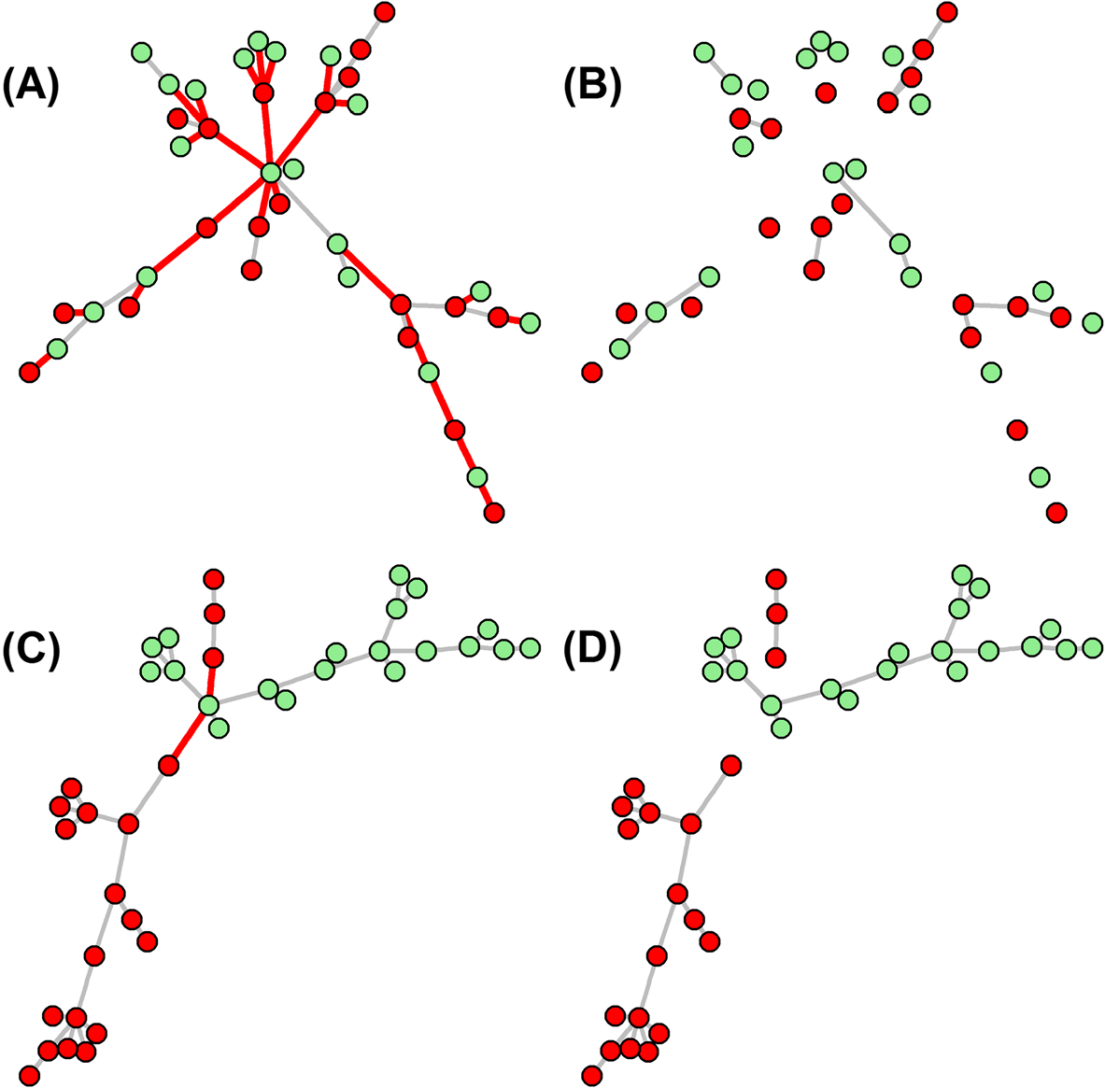
Two illustrative examples of the disjoint MST subtrees. (1) The MST of the pooled samples of *X*, *Y ~ N*(0_*p*×1_,*I*_*p×p*_) (*H*_0_ is true) is shown in panel **a** and its 27 disjoint subtrees (*R* = 27) are shown in panel **b**; (2) The MST of the pooled samples of *X ~ N*(0_*p*×1_,*I*_*p×p*_) and *Y ~ N*(1_*p*×1_,*I*_*p×p*_) (*H*_1_ is true) is shown in panel **c** and its 3 disjoint subtrees (*R* = 3) are shown in panel **d**

Figure 3 presents two illustrative examples: (1) The MST of the pooled samples of *X*, *Y ~ N*(**0**_*p*×1_,*I*_*p×p*_) (*H*_0_ is true) is shown in panel A and its disjoint subtrees (*R* = 27) are shown in panel B; (2) The MST of the pooled samples of *X ~ N*(**0**_*p*×1_,*I*_*p×p*_) and *Y ~ N*(**1**_*p*×1_,*I*_*p×p*_) (*H*_0_ is false) is shown in panel C and its disjoint subtrees (*R* = 3) are shown in panel D. **0**_*p*×1_ and **1**_*p*×1_ are *p*-dimensional mean vectors of zeros and ones, and *I*_*p*×*p*_ is the *p* × *p* identity matrix. Applying function WWtest to these two cases yields *P*-value = 0.813 and *P*-value < 0.001, respectively.

### Multivariate Kolmogorov-Smirnov and mean deviation tests

The vertices in the MST are ranked based on a specific scheme and the test statistic is calculated based on these ranks. Package GSAR supports two statistics: (1) The Kolmogorov-Smirnov (KS) statistic which calculates the maximum deviation between the Cumulative Distribution Functions (CDFs) of the ranks between *X* and *Y* samples, i.e., the maximum absolute difference between the number of observations from $X$ and $Y$ ranked lower than *i*, 1 ≤ *i* ≤ N, is the test statistic [10, 29]

$$ D=\sqrt{\frac{n_1{n}_2}{n_1+{n}_2}}\ \underset{i}{ \max}\left|{d}_i\right| $$

where

$$ {d}_i=\frac{r_i}{n_1}-\frac{s_i}{n_2} $$

and *r*_*i*_(*s*_*i*_) is the number of vertices (observations) in *X*(*Y*) ranked lower than *i*; (2) The mean deviation (MD) statistic which calculates the average deviation between the CDFs of the ranks between *X* and *Y* samples. The MD statistic for a gene set of size *p* is defined as

$$ D={\displaystyle \sum_{i=1}^p}\left[P\left(X,i\right)-P\left(Y,i\right)\right] $$

where

$$ P\left(X,i\right)=\frac{{\displaystyle {\sum}_{j\in X,j\le i}}\ {r}_j^{\alpha }}{{\displaystyle {\sum}_{j\in X}}\ {r}_j^{\alpha }} $$

and

$$ P\left(Y,i\right)={\displaystyle \sum_{j\in Y,j\le i}}\frac{1}{n_2} $$

*r*_*j*_ is the rank of sample *j* in the MST and the exponent *α* is set to 0.25 to give the ranks a modest weight. Although the MD statistics use sample ranks here, a similar statistic that calculates the average deviation of CDFs of gene ranks between a gene set and its complement has been used successfully in the context of single sample gene set enrichment analysis [5, 31]. For both KS and MD statistics, the null distribution is estimated by permuting sample labels and calculating the statistic *D* for a large number of times *M*. While the MD statistic has asymptotically a normal distribution and tests a two-sided hypothesis, the KS statistic asymptotically follows the Smirnov distribution [29] and tests a one-sided hypothesis. Hence the *P*-values for these two methods are estimated as

$$ P- valu{e}_{MD}=\frac{{\displaystyle {\sum}_{k=1}^M}\kern0.5em I\left[\left|{D}_k\right|\ge \left|{D}_{obs}\right|\right]+1}{M+1} $$

$$ P- valu{e}_{KS}=\frac{{\displaystyle {\sum}_{k=1}^M}\kern0.5em I\left[{D}_k\ge {D}_{obs}\right]+1}{M+1} $$

Sample ranking scheme in the MST can be designed to confine a specific alternative hypothesis more power. Two alternatives are currently considered in GSAR. First, functions KStest and MDtest test the null hypothesis ${H}_0:\ {\overline{\mu}}_X={\overline{\mu}}_Y$ against the alternative ${H}_1:\ {\overline{\mu}}_X\ne {\overline{\mu}}_Y$. The MST is rooted at a vertex with the largest geodesic distance (i.e., one of two vertices that form the ends of the longest path in the tree) and the rest of the vertices are ranked according to the high directed preorder (HDP) traversal of the tree [10, 29]. Function HDP.ranking in package GSAR returns the vertices ranks in a MST according to the HDP traversal. Second, the radial Kolmogorov-Smirnov (function RKStest) and radial mean deviation (function RMDtest) methods test the null hypothesis *H*_*0*_: *var*(*X*) *≠ var*(*Y*) against the alternative *H*_1_: *var*(*X*) *≠ var*(*Y*) or equivalently ${H}_0:\ {\overline{\sigma}}_X={\overline{\sigma}}_Y$ against ${H}_1:\ {\overline{\sigma}}_X\ne {\overline{\sigma}}_Y$ where ${\overline{\sigma}}_X$ and ${\overline{\sigma}}_Y$ are respectively the standard deviations of *X* and *Y*. The MST is rooted at the vertex of smallest geodesic distance (centroid) and vertices are ranked based on their depth and distance from the root such that ranks are increasing radially from the root (function radial.ranking). Although the power analysis in [10] showed that the radial ranking scheme provides the differential variance hypothesis with higher detection power than the differential mean hypothesis, yet the power of detecting the latter alternative is non-negligible. To attain higher confidence in the results of RKS or RMD methods, they can be supplemented by KS or MD methods and then only the gene sets that satisfy: *P*-value_RKS_ < *α* and *P*-value_KS_ > *α* (or *P*-value_RMD_ < *α* and *P*-value_MD_ > *α*) should be considered. Then the null will be rejected when the alternative ${H}_1:\ {\overline{\sigma}}_X\ne {\overline{\sigma}}_Y$ is true but not the alternative ${H}_1:\ {\overline{\mu}}_X\ne {\overline{\mu}}_Y$. The following R commands implement the KS, MD, RKS, and RMD methods

KStest(object, group, nperm = 1000, pvalue.only = TRUE)

MDtest(object, group, nperm = 1000, pvalue.only = TRUE)

RKStest(object, group, mst.order = 1, nperm = 1000, pvalue.only = TRUE)

RMDtest(object, group, mst.order = 1, nperm = 1000, pvalue.only = TRUE)

where object is a numeric matrix of gene expression with columns and rows corresponding to samples and genes, respectively, group is a numeric vector (with values 1 and 2) indicating group associations for samples, nperm is the number of permutations used to estimate the null distribution, mst.order is a numeric value indicating the number of MSTs considered in the radial ranking procedure (see the union of MSTs subsection below for further details), and pvalue.only is a logical parameter that indicates if returning the *P*-value only is desired. When pvalue.only = FALSE, the observed statistic, the vector of permuted statistics, and the *P*-value are returned in a list (see Additional file 2 for examples of real dataset analysis).

**Fig. 4:**
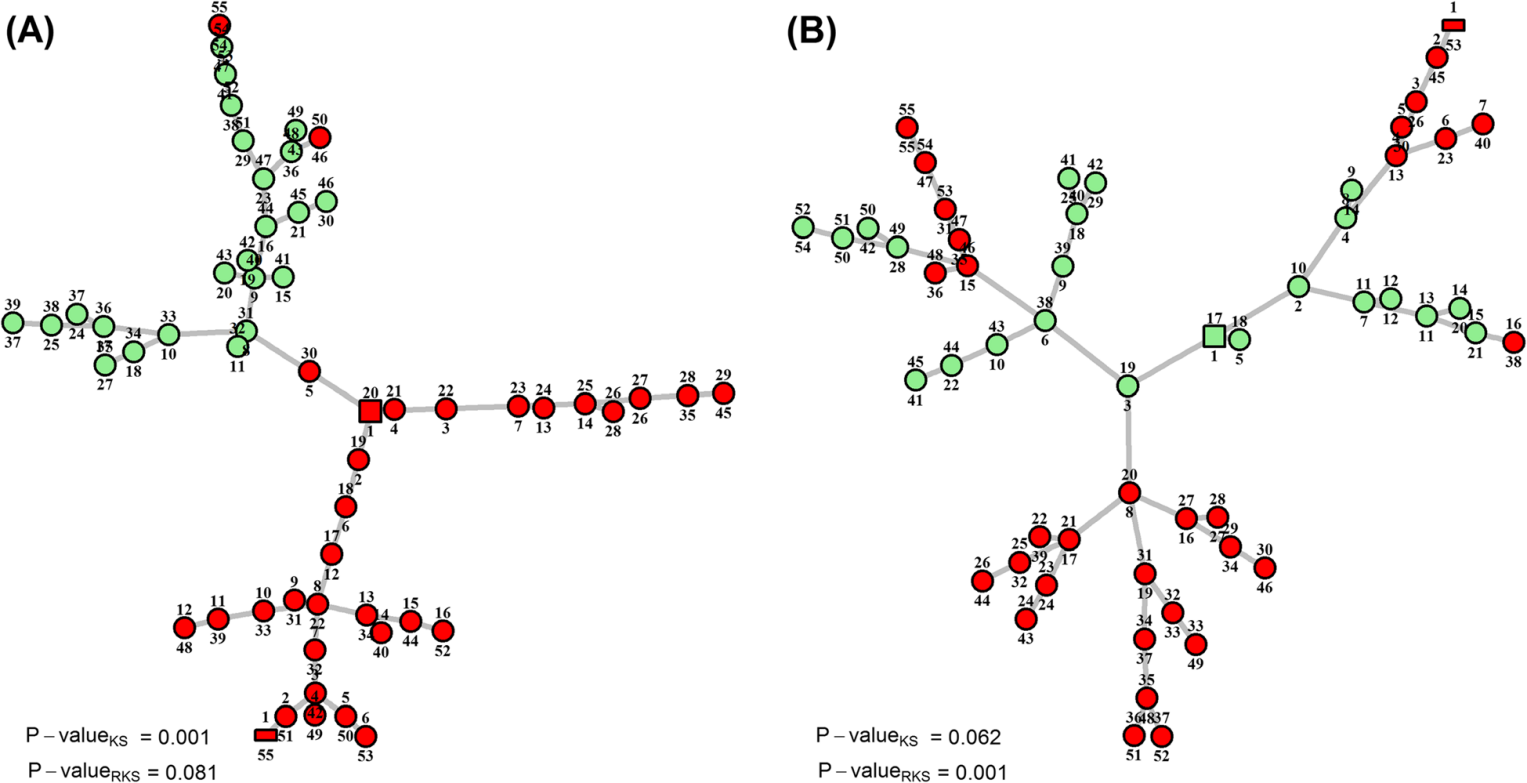
Two illustrative examples using 23 normal samples and 32 clear cell renal cell carcinoma samples from the GSE15641 dataset. **a** The MST of the pooled normal and tumor samples considering 67 genes from the *KEGG renal cell carcinoma* gene set. The samples of each phenotype are grouped together in the tree and the KS test rejects the null (${H}_1:\ {\overline{\mu}}_X\ne {\overline{\mu}}_Y$ is true) but not RKS test; **b** The MST of the pooled normal and tumor samples considering 19 genes from the *KEGG glycosylphosphatidylinositol (GPI) anchor biosynthesis* gene set. Normal samples constitute the backbone of the MST while tumor samples form the branches and RKS test rejects the null (${H}_1:\ {\overline{\sigma}}_X\ne {\overline{\sigma}}_Y$ is true) but not KS test. The roots of the HDP and radial ranking schemes in the MSTs are highlighted with rectangle and square shapes, respectively

Figure 4 presents two illustrative examples using normal (23 samples) and clear cell renal cell carcinoma (32 samples) samples from a real gene expression dataset [32] that is available from the gene expression omnibus repository (accession number GSE15641). Selected gene sets from the Kyoto encyclopedia of genes and genomes (KEGG) [33] were obtained from the curated collection of the molecular signatures database (MSigDB) [34]. The MST of the pooled normal and tumor samples, considering 67 genes from the KEGG ‘renal cell carcinoma’ gene set is shown in panel A. The samples of each phenotype are grouped together in the tree, suggesting separation between the two phenotypes in *R* ^*p*^. The KS test rejects the null hypothesis (${H}_1:\ {\overline{\mu}}_X\ne {\overline{\mu}}_Y$ is true) while the RKS test fails to do so. The MST of the pooled samples, considering 19 genes from the KEGG ‘Glycosylphosphatidylinositol anchor biosynthesis’ gene set is shown in panel B. Normal samples constitute the backbone of the MST while tumor samples form the branches. The centroid vertex in the MST naturally occupies the center of the backbone and hence the difference in ranks is large between the two phenotypes. The RKS test rejects the null hypothesis (${H}_1:\ {\overline{\sigma}}_X\ne {\overline{\sigma}}_Y$ is true), while the KS test fails. The HDP and radial rankings of vertices in the MST are shown above and below the vertices in both panels. While most vertices are represented by circles, the roots of the HDP and radial rankings are highlighted as rectangular and square shapes, respectively.

### Aggregated F-test of variance

The univariate *F*-test is used to find differential variability in individual genes similar to [12]. The *F*-statistic for gene *i*, ${F}_i={\overline{\sigma}}_{X_i}/{\overline{\sigma}}_{Y_i}$, represents the ratio between the phenotype variances and follows the *F*-distribution with *n*_1_-1 and *n*_2_-1 degrees of freedom when the null hypothesis ${H}_0:{\overline{\sigma}}_{X_i}={\overline{\sigma}}_{Y_i}$ is true for gene *i*. The null hypothesis is rejected if *F*_*i*_ is too large or too small. Then individual *P*-values for the genes in a gene set are aggregated to obtain a score statistic. Comparisons among aggregation methods such as Fisher’s probability combining method, Stouffer’s method, and Gamma method in the context of DE analysis determined that Fisher’s method performed best in terms of power and Type I error rate [9, 35]. Assigning all individual *P*-values (*P*_*i*_, 1 ≤ *i* ≤ *p*) equal weights, function aggrFtest uses Fisher’s method to calculate the aggregated test statistic [36]

$$ T=-2{\displaystyle \sum_{i=1}^p}lo{g}_e\left({P}_i\right)=-2\ lo{g}_e\left({\displaystyle \prod_{i=1}^p}{P}_i\right) $$

When all *P*-values are independent, the test statistic *T* follows the Chi-square distribution with 2*p* degrees of freedom. Since independence assumption is often violated for expression data, significance is estimated by permuting sample labels and calculating *T* many times (*M*). *P*-value is the proportion of permutations yielding equal or more extreme statistic than the one obtained from the original observed data, i.e.,

$$ P- valu{e}_{aggrFtest}=\frac{{\displaystyle {\sum}_{k=1}^M}I\left[{T}_k\ge {T}_{obs}\right]+1}{M+1} $$

This method tests the null hypothesis that all genes in the gene set show no differential variance between two conditions against the alternative hypothesis that at least one gene shows differential variance, i.e., the null $\forall i:\ {\overline{\sigma}}_{X_i}={\overline{\sigma}}_{Y_i}$ where 1 ≤ *i ≤ p* against the alternative $\exists i:\ {\overline{\sigma}}_{X_i}\ne {\overline{\sigma}}_{Y_i}$. The following R command implements the method

AggrFtest(object, group, nperm = 1000, pvalue.only = TRUE)

The command parameters are exactly the same as defined above for other methods.

### Gene sets net correlations analysis

The GSNCA method detects the differences in net correlation structure for a gene set between two conditions [20] and is implemented in function GSNCAtest. The genes under each phenotype are assigned weight factors which are adjusted simultaneously such that equality is achieved between each gene’s weight and the sum of its weighted correlations with other genes in a gene set of *p* genes

$$ {w}_i={\displaystyle \sum_{j\ne i}}{w}_j\ \left|{r}_{ij}\right|,\kern0.75em 1\le i\le p $$

where *r*_*ij*_ is the correlation coefficient between genes *i* and *j*. The problem is solved as an eigenvector problem with a unique solution which is the eigenvector corresponding to the largest eigenvalue of the genes’ correlation matrix [20]. The test statistic *w*_*GSNCA*_ is the first norm between the two scaled weight vectors under two phenotypes where each vector is multiplied by its norm. This statistic tests the null hypothesis *H*_0_ : *w*_*GSNCA*_ = 0 against the alternative *H*_1_ : *w*_*GSNCA*_ ≠ 0 and detects changes in the intergene correlations structure between two phenotypes. This test differs from other methods like GSCA and dCoxS that detect any changes in the correlation matrix, i.e., *H*_0_ : *C*_*X*_ = *C*_*Y*_ against the alternative *H*_1_ : *C*_*X*_ ≠ *C*_*Y*_, in the sense that it detects how correlations change relative to each other. For example, when *C*_*X*_ = *a C*_*Y*_ and *a* is constant, eigenvectors of of *C*_*X*_ and *C*_*Y*_ are identical, however they have different eigenvalues and average difference in pairwise correlations between the two conditions. Hence GSCA detects differential correlation but GSNCA does not detect change in the net correlation structure. The following R command implements the method (see Additional file 2 for examples of real dataset analysis)

GSNCAtest(object, group, nperm = 1000, cor.method = "pearson", check.sd = TRUE, min.sd = 1e-3, max.skip = 10, pvalue.only = TRUE)

where check.sd is a logical parameter indicating if the standard deviations of gene expressions should be checked for small values before intergene correlations are computed, min.sd the minimum allowed standard deviation for any gene in the gene set where execution stops and an error message is returned if the condition is violated, max.skip is maximum number of skipped random permutations which yield any gene with a standard deviation less than min.sd, and cor.method is a character string indicating which correlation coefficient is used to calculate intergene correlations (Pearson, Spearman or Kendall). The rest of the parameters are exactly the same as defined earlier for other methods.

The need to guard against zero standard deviation arises in the case of RNA-seq count data where non-expressed genes may yield zero counts across most samples and produce zero or tiny standard deviation for one or more genes in the gene set. Such situation produces an error while computing the correlation coefficients between genes. When check.sd = TRUE, standard deviations are checked in advance and if any is smaller than min.sd (default is 10^-3^), the execution stops and an error message is returned indicating the number of feature causing the problem. Another similar problem arises when non-expressed genes yield zero counts across some samples under two phenotypes. Permuting sample labels may group such zero counts under one phenotype by chance and produce a standard deviation smaller than min.sd. To allow the method to skip such permutations without causing excessive delay, an upper bound is set for the number of allowed skips (max.skip). If the upper limit is exceeded, an error message is returned.

### The union of MSTs

The second MST is defined as the MST of the full network after excluding the links of the first MST, i.e., the subgraph *G*(*V*,*E*-*T*_1_). Package GSAR provides function findMST2 to find the union of the first and second MSTs (referred to by MST2). The wrapper function plotMST2.pathways plots the MST2 of a gene set under two conditions side-by-side to facilitate the comparison between the correlation structure and hub genes. A gene with high intergene correlations in the set tends to occupy a central position and has relatively high degree in the MST2 because the shortest paths connecting the vertices of the first and second MSTs pass through such gene. In contrast, a gene with low intergene correlations occupies a non-central position and has low degree (typically 2). This property of the MST2 makes it a valuable visualization tool to examine the full correlation network by highlighting the most highly correlated genes. We illustrate the MST2 approach by considering selected gene sets from the p53 dataset.

**Fig. 5:**
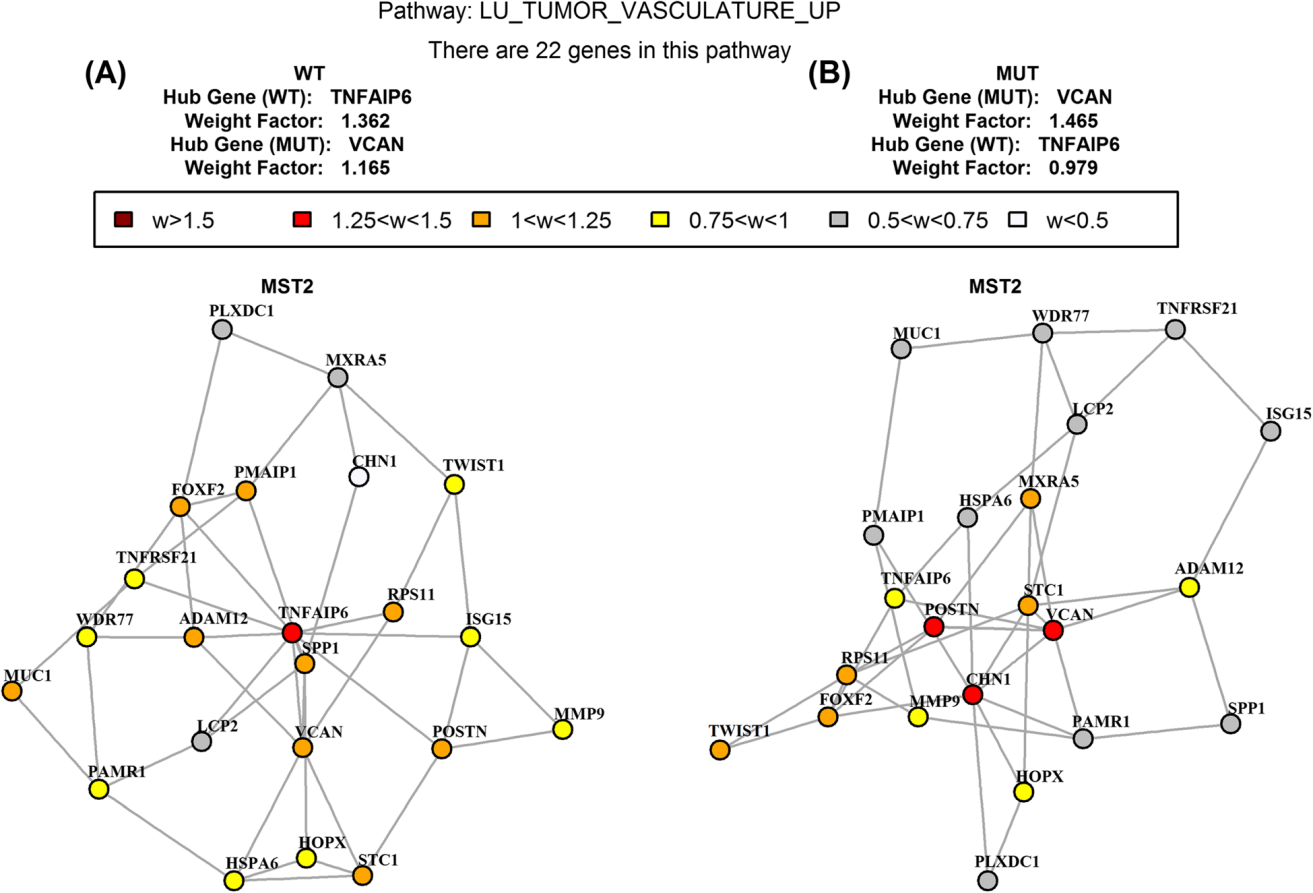
MST2 of the *Lu tumor vasculature up* gene set obtained from the C2 collection of curated gene sets in MSigDB. **a** wild type p53 samples show TNFAIP6 as the hub gene; **b** mutated p53 samples show VCAN as the hub gene

Figure 5 shows the MST2 of the ‘Lu tumor vasculature up’ gene set obtained from the C2 collection (version 3.0) of curated gene sets in the MSigDB [34]. This gene set consists of genes over-expressed in ovarian cancer endothelium and was detected by GSNCA (*P*-value < 0.05) but not by GSCA (*P*-value > 0.05) [20]. The MST2 in Fig. 5a (wild type p53) identifies gene TNFAIP6 (tumor necrosis factor, *α*-induced protein 6) as a hub genes. This gene was found to be 29.1 fold over-expressed in tumor endothelium, and was suggested to be specific for ovarian cancer vasculature [37]. Identifying TNFAIP6 as a hub gene in this gene set suggests that it could be an important regulator of ovarian cancer and supports the original observation. The MST2 in Fig. 5b (mutated p53) identifies gene VCAN (Versican) as a hub gene. VCAN is involved in cell adhesion, proliferation, angiogenesis and plays a central role in tissue morphogenesis and maintenance and its increased expression is observed for tumor growth in multiple tissue types [38, 39]. This gene contains p53 binding sites and its expression correlates with p53 dosage [40]. Hence, the role of both hub genes identified by MST2 (TNFAIP6 and VCAN) and previous findings in the literature support identifying them as hubs by the MST2 and indicates the usefulness of MST2 in provide information regarding the underlying biological processes in well-defined gene sets.

**Fig. 6:**
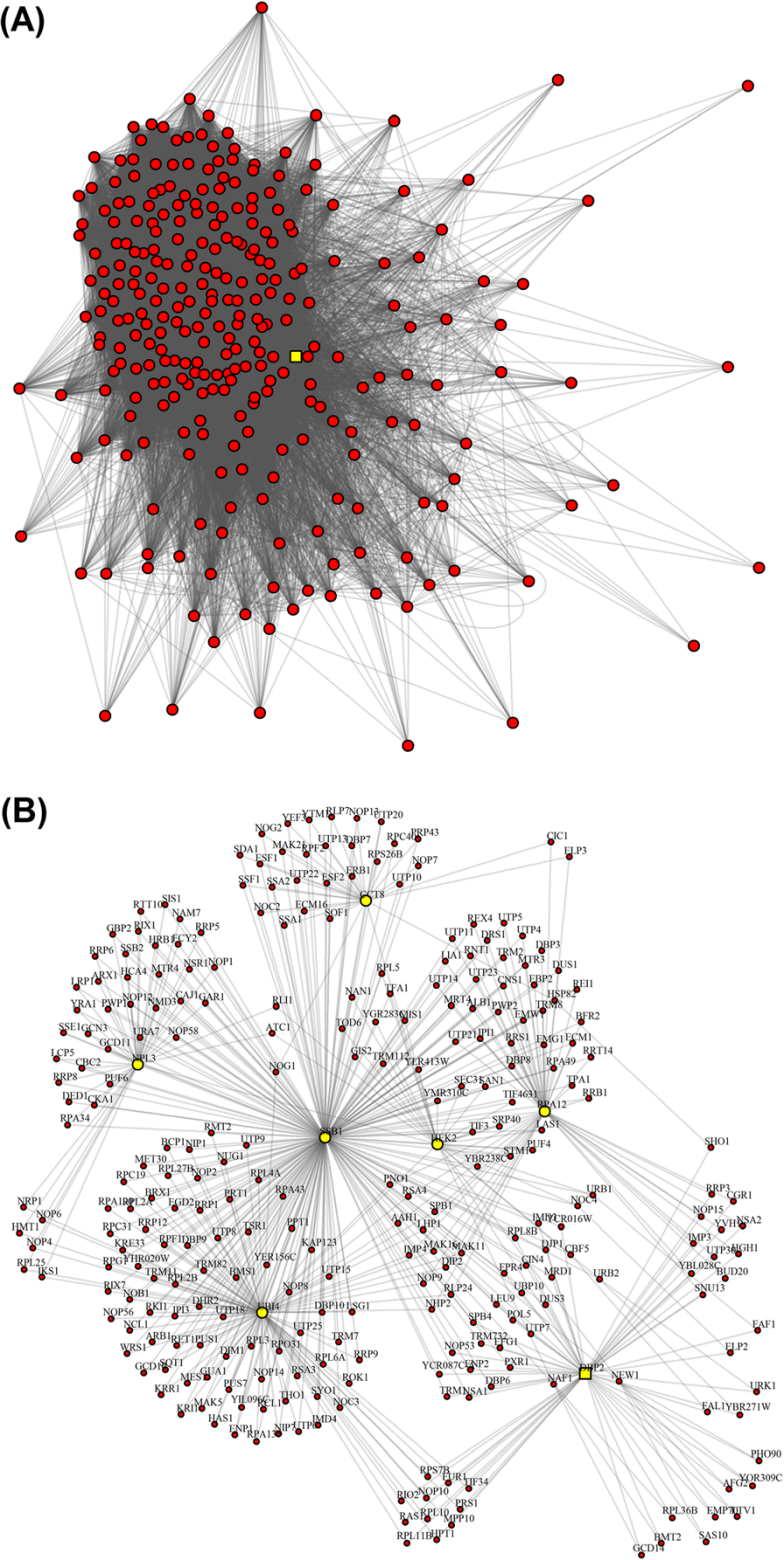
The yeast PPI network constructed using information retrieved from PINA and String databases of interactions. **a** first-degree neighborhoods network around the DBP2 yeast helicase; **b** the respective derived MST2. While the network of first-degree neighborhoods appears crowded in disorderly manner, the corresponding MST2 representation reveals fine network structure with highly connected molecular chaperons, protein modifiers and regulators occupying central positions

In addition to gene expression data, MST2 can be informative in deciphering the properties of protein-protein interaction (PPI) networks by highlighting the minimum set of essential interactions among proteins. PPI networks can be represented by graphs with undirected binary edges and the adjacency matrix is used here instead of the correlation matrix to find the MST2. Figure 6 (reproduced with permission from [41]) shows the yeast PPI network constructed using information retrieved from PINA [42] and String [43] databases of interactions. Panel A shows the first-degree neighborhoods around the DBP2 yeast helicase and panel B shows its MST2. While the full network of first-degree neighborhoods appears crowded and disordered, the corresponding MST2 representation reveals fine network structure with highly connected molecular chaperons, protein modifiers and regulators occupying central positions (e.g., UBI4 and SSB1) [41].

Functions RKStest and RMDtest can also use the union of the first *k* MSTs (1 < *k* ≤ 5) instead of the first MST to include more links before performing their ranking procedure to achieve higher detection power. Generally, power gain diminishes when *k* > 3 and including higher order MSTs achieves no benefit.

### Computational considerations

Non-parametric methods have longer execution time as compared to parametric methods, however they are necessary whenever distributional assumptions are violated. Additional file 1: Table S2 provides an assessment of the execution times expected when selected methods from package GSAR are used with different sample size and gene set size parameters. Additional file 1 also presents a simple example with R code performing parallel computing of a selected group of gene sets from the first case study in Additional file 2. Parallel computing of large groups of gene sets reduces execution times significantly and is possible whenever multiple core machines or high performance computing (HPC) facilities are accessible.

## Conclusions

Bioconductor package GSAR provides a set of statistical methods for analyzing omics datasets. The package also implements a convenient graphical visualization tool to aid in deciphering the hidden structures in complex networks. The methods in package GSAR are applicable to any type of omics data that can be represented in a matrix format.

## Additional files

Additional file 1: Additional document presenting computational considerations and uniqueness of package GSAR. (DOCX 32 kb)
Additional file 2: Vignette of package GSAR. (PDF 732 kb)
Additional file 3: Source file ‘GSAR_1.9.1.tar.gz’. (GZ 2174 kb)

## Abbreviations

DC: Differential co-expression
DE: Differential expression
DV: Differential variability
GSA: Gene set analysis
GSCA: Gene set co-expression analysis
GSEA: Gene set enrichment analysis
GSNCA: Gene sets net correlation analysis
KEGG: Kyoto Encyclopedia of genes and genomes
KS: Kolmogorov-Smirnov
MD: Mean deviation
MSigDB: Molecular signature database
MST: Minimum spanning tree
MUT: Mutated
PPI: Protein-protein interaction
RKS: Radial Kolmogorov-Smirnov
RMD: Radial mean deviation
WT: Wild type
WW: Wald-Wolfowitz
