# Supplementary material for: GSAR: Bioconductor package for Gene Set analysis in R
Source: BMC Bioinformatics. 2017 Jan 24;18:61. doi: 10.1186/s12859-017-1482-6 (PMC5259853; doi:10.1186/s12859-017-1482-6)
Supplement: Additional file 1: — Additional document presenting computational considerations and uniqueness of package GSAR. (DOCX 32 kb) [file 12859_2017_1482_MOESM1_ESM.docx]

| *(Additional file 1)*  **GSAR: Bioconductor package for Gene Set Analysis in R**  Yasir Rahmatallah^1,*^, Boris Zybailov^2^, Frank Emmert-Streib^3^ and Galina Glazko^1^  ^1^Department of Biomedical Informatics, University of Arkansas for Medical Sciences, Little Rock, AR 72205.  ^2^Department of Biochemistry and Molecular Biology, University of Arkansas for Medical Sciences, Little Rock, AR 72205.  ^3^Computational Medicine and Statistical Learning Laboratory, Tampere University of Technology, Korkeakoulunkatu 1, Tampere, Finland FI-33720.  * Corresponding author. |
| --- |

**Uniqueness of Package GSAR**

There are many available packages that implement different gene set analysis (GSA) methods. Some of these implement competitive and others implement self-contained methods. Table S1 lists a representative set of available gene set analysis methods. Competitive approaches consist mainly of gene set enrichment analysis (GSEA) methods that compare a gene set in two conditions against its complement which consists of all the genes in the dataset excluding the genes in the set itself. Self-contained approaches test if a gene set is expressed differently, e.g. differential expression (DE), differential variance (DV), or differential co-expression (DC), between two experimental conditions, regardless of the rest of the genes in the dataset. Self-contained methods are more flexible in testing different hypotheses as compared to competitive methods. The methods implemented in package GSAR are all self-contained and are comparable to other self-contained methods that test similar specific alternative hypothesis. There are two advantages that distinguish package GSAR:

First, package GSAR tests multiple specific alternative hypotheses, including the multivariate differential variance. While some methods such as N-statistic test a general hypothesis and lack specificity (detect differences in both mean and variance), others like ROAST and SAM-GS test a specific alternative hypothesis (detect differential mean only). Methods for DV gene set analysis are non-existing to the best of our knowledge. Implementing the RMDtest and RKStest functions in GSAR is an attempt to fill this gap that offers a unique advantage when testing DV of gene sets is desired.

Second, pairing the GSNCA method with MST2 visualization offers convenience by highlighting key genes in the gene set based on co-expression patterns, and independently on MST2. Plotting the MST2 of a gene set under two conditions side-by-side shows the changes in correlation structure, and highlights hub genes (see Figure 5). MST2 is also informative in deciphering the properties of protein-protein interaction (PPI) networks by highlighting the minimum set of essential interactions among proteins, that are retrieved from databases or experiments (see Figure 6). In general, MST2 is a valuable visualization tool to examine the spatial properties of nodes in a network.

**Table S1.** A representative set of available methods for gene set analysis.

| Method | Availability | Self-contained/ Competitive | Parametric? | Multivariate/  Aggregation | Alternative hypothesis |
| --- | --- | --- | --- | --- | --- |
| ROAST | limma (Bioconductor) | Self-contained | Yes | Multivariate | $\bar{\mu}_{X}\neq\bar{\mu}_{Y}$ (DE) |
| N-statistic | cramer (CRAN) | Self-contained | No | Multivariate | $F_{X}\neq F_{Y}$ |
| SAM-GS | Code available at  <https://sites.ualberta.ca/~yyasui/software.html#SAM-GS> | Self-contained | No | Aggregation | $\bar{\mu}_{X}\neq\bar{\mu}_{Y}$ (DE) |
| GSCA | Code available at  <https://www.biostat.wisc.edu/~kendzior/GSCA> | Self-contained | No | Aggregation | $C_{X}\neq C_{Y}$ (DC) |
| CoGA | Package available at  <https://www.ime.usp.br/~suzana/coga/index> | Self-contained | No | Multivariate | $C_{X}\neq C_{Y}$ (DC) |
| runDecode | DECODE (CRAN) | Self-contained | Yes | Aggregation | $\bar{\mu}_{X}\neq\bar{\mu}_{Y}$ (DE) $C_{X}\neq C_{Y}$ (DC) |
| ROMER | limma (Bioconductor) | Competitive (supervised gene set enrichment analysis) | | | |
| PGSEA | PGSEA (Bioconductor) | Competitive (supervised gene set enrichment analysis) | | | |
| R-GSEA | Code available at  <http://software.broadinstitute.org/gsea/downloads.jsp> | Competitive (supervised gene set enrichment analysis) | | | |
| GSVA | GSVA (Bioconductor) | Competitive (unsupervised gene set enrichment analysis) | | | |
| ssGSEA | GSVA (Bioconductor) | Competitive (unsupervised gene set enrichment analysis) | | | |

**Execution Times**

Non-parametric methods rely on permuting sample labels to estimate significance and require longer execution time compared to parametric methods. They are however necessary when distributional assumptions do not apply. Execution time is usually proportional to the number of permutations performed to estimate the null distribution of the test statistic. It is widely acceptable to use 1000 permutations to estimate *P*-values and performing 10000 permutations mostly affects only marginal *P*-values that are close to the selected significance level. Table S2 presents execution times (in seconds) of selected methods in package GSAR for different sample size (*N*) and gene set size (*p*) values. We used 1000 permutations for each method and averaged the execution time over 100 iterations to obtain stable estimates. While the number of permutations affects execution time for all methods uniformly, sample size and gene set size affect different methods differently. For example, a significant fraction of the computational burden in functions KStest, RKStest, MDtest, and RMDtest is spent on finding the MST and ranking its *N* nodes, and a tiny fraction is spent on calculating the Euclidean distance between the *p*-dimensional samples. Since nodes correspond to samples, more samples means larger MST and longer ranking list, hence sample size affects the execution time significantly, while gene set size effect is virtually negligible (see Table S2). Another example is function GSNCAtest where a significant fraction of the computational burden is spent on solving the eigen decomposition problem for a *p*×*p* correlation matrix and a tiny fraction is spent on calculating the correlation coefficients between genes. Therefore, gene set size (*p*) affects the execution time significantly, while sample size (*N*) effect is very small (see Table S2).

**Table S2.** Execution time (in seconds) averaged over 100 iterations of different methods performed with 1000 permutations for different sample size (*N*) and gene set size (*p*) values.

| KS | RMD | Sample Size | | | | | |
| --- | --- | --- | --- | --- | --- | --- | --- |
| WW | **GSNCA** | ***N*=20** | | ***N*=40** | | ***N*=60** | |
| Gene Set Size | ***p*=10** | 0.28 | 0.27 | 0.67 | 0.67 | 1.09 | 1.14 |
|  |  | 5.62 | 0.45 | 7.26 | 0.45 | 8.81 | 0.47 |
|  | ***p*=50** | 0.26 | 0.26 | 0.58 | 0.64 | 1.04 | 1.14 |
|  |  | 5.39 | 1.52 | 7.08 | 1.58 | 8.91 | 1.62 |
|  | ***p*=100** | 0.27 | 0.27 | 0.59 | 0.64 | 1.04 | 1.17 |
|  |  | 5.48 | 5.85 | 7.16 | 5.93 | 9.10 | 6.19 |

**Parallel Processing of Gene Sets**

Sometimes it is desired to perform one or more statistical methods for a large number of gene sets. Performing such computations in a sequential manner may take a long time. One possible solution to reduce execution times is to perform parallel computing whenever multiple core machines or high performance computing (HPC) facilities are accessible. The simplest possible way to achieve this goal is to create a common function to perform the desired computations, divide the large group (or list) of gene sets into smaller subgroups, and assign each individual core one subgroup to perform the common function. The R code shown below performs the GSNCA method for the first 100 C2 gene sets that were obtained in the first case study in Additional file 2. After executing the code of the first case study (see Additional file 2), the code below can be used. The code defines a common function that performs the GSNCA method, divides the list of 100 gene sets into four smaller lists, and employs four cores to perform the parallel computations, where each individual core is assigned one list of 25 gene sets. Command mcapply from R package parallel is used in this example. The returned value results by command mclapply is a list object of length four, where each item consists of a numeric vector of 25 *P*-values returned by function fn. The final outcome pvalues is a numeric vector of 100 *P*-values, named by their corresponding C2 gene set names.

fn <- function(pathlist.item)

{

vec_pvalue <- array(1, c(1,length(pathlist.item)))

names(vec_pvalue) <- names(pathlist.item)

vec_pvalue <- TestGeneSets(object=p53DataSet, group=group.label,

geneSets=pathlist.item, test="GSNCAtest")

return(unlist(vec_pvalue))

}

library(parallel)

pathlist <- list(c2.pathways[1:25], c2.pathways[26:50], c2.pathways[51:75], c2.pathways[76:100])

results <- mclapply(X=pathlist, FUN=fn, mc.cores=4, mc.preschedule=TRUE, mc.set.seed=TRUE, mc.cleanup=TRUE)

pvalues <- unlist(results)
