# Supplementary material for: GSAR: Bioconductor package for Gene Set analysis in R
Source: BMC Bioinformatics. 2017 Jan 24;18:61. doi: 10.1186/s12859-017-1482-6 (PMC5259853; doi:10.1186/s12859-017-1482-6)
Supplement: Additional file 3: — Source file ‘GSAR_1.9.1.tar.gz’. (GZ 2174 kb) [file 12859_2017_1482_MOESM3_ESM.gz › GSAR/inst/doc/GSAR.pdf]

Gene Set Analysis in R – the GSAR Package

Yasir Rahmatallah[1] and Galina Glazko[2]
Department of Biomedical Informatics,
University of Arkansas for Medical Sciences,
Little Rock, AR 72205.
[1]yrahmatallah@uams.edu, [2]gvglazko@uams.edu

GSAR version 1.9.1 (Last revision 2016-08-08)

Contents

1 Introduction **2**

2 Minimum spanning trees **3**
2.1 First MST 3
2.2 MST2 for correlation and PPI networks 4

3 Statistical methods **4**
3.1 Wald-Wolfowitz test 4
3.2 Kolmogorov-Smirnov tests 5
3.3 Mean deviation tests 6
3.4 Aggregated F-test 7
3.5 Gene sets net correlations analysis 7
3.5.1 Method 7
3.5.2 The problem of zero standard deviation 8

4 Notes on handling RNA-seq count data **8**

5 Case studies **8**
5.1 The p53 dataset 8
5.1.1 Introduction 8
5.1.2 Filtering and normalization 9
5.1.3 GSA 9
5.2 The ALL dataset 12
5.2.1 Introduction 12
5.2.2 Filtering and normalization 12
5.2.3 Selected gene set 13
5.3 The Pickrell dataset 14
5.3.1 Introduction 14
5.3.2 Filtering and normalization 16
5.3.3 Testing selected pathways 17

6 Session info **18**

1 Introduction

This vignette provides an overview of the *R* package *GSAR* which provides a set of multivariate statistical tests for self-contained gene set analysis (GSA). *GSAR* consists of two-sample multivariate nonparametric statistical methods testing a null hypothesis against specific alternative hypotheses, such as differences in mean (shift), variance (scale) or correlation structure. It also offers a graphical visualization tool for the correlation networks obtained from expression data to examine the change in the net correlation structure of a gene set between two conditions based on the minimum spanning trees. The same tool also works for protein-protein Interaction (PPI) networks to highlight the most influential proteins. The package implements the methods proposed in [1, 2, 3] which were thoroughly tested using simulated and microarray datasets in [1] and [2]. Figure 1 shows the outline of the package. While the test functions in the package analyze one gene set at a time, the wrapper function `TestGeneSets` receives a list of gene sets and invokes a specific test function for all the gene sets in a sequential manner.

The methods in the package can also be applied to RNA-seq count data given that proper normalization which accounts for both the within-sample differences (gene lengths) and between-samples differences (library sizes) is used. However, for RNA-seq data the variance is a function of mean expression and applying the variance tests (`RKStest`, `RMDtest`, `AggrFtest`) to RNA-seq data highly depends on the normalization method. This specific topic is not well-studied and characterized yet. This vignette begins with a brief overview of the theoretical concepts behind the statistical methods, and then provides a number of fully worked case studies, from microarrays to RNA-seq count data.

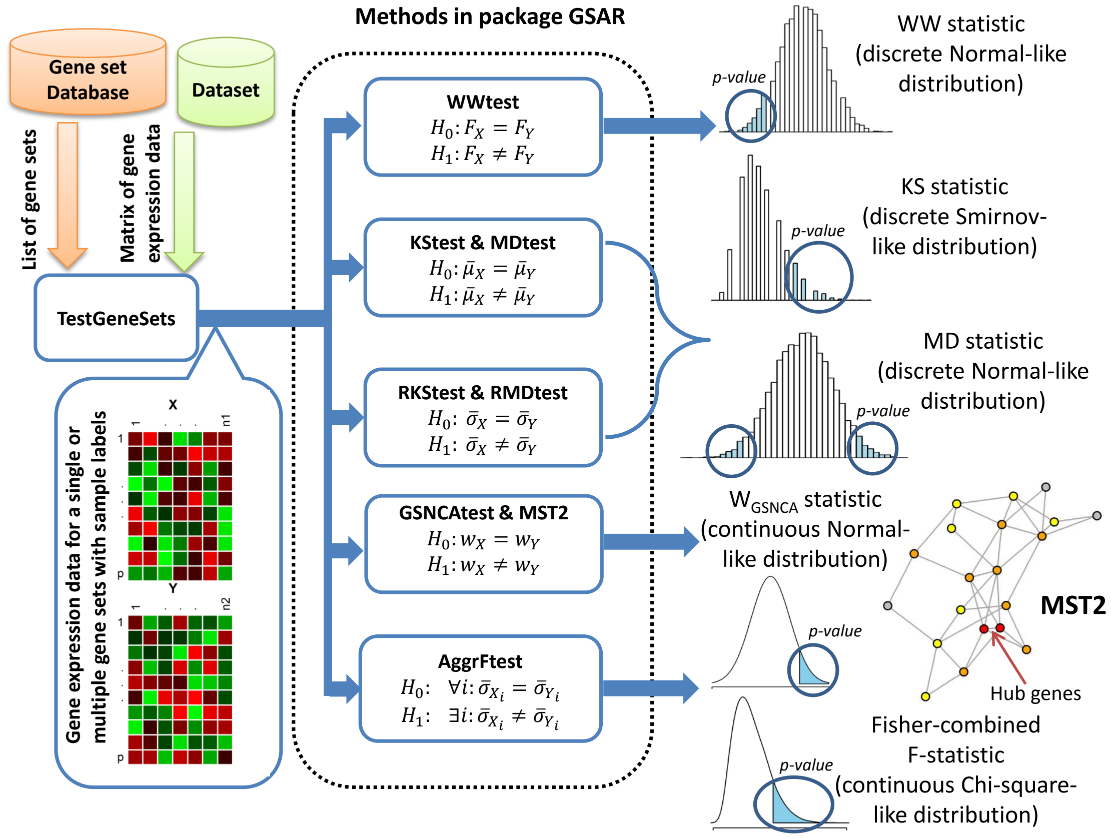

Figure 1: GSAR package outline. The inputs for the statistical tests can be (1) the matrix of gene expression for a single gene set in the form of normalized microarray or RNA-seq data and a vector of labels indicating to which condition each sample belongs; or (2) the matrix of gene expression for all genes, a vector of labels indicating to which condition each sample belongs, and a list of gene sets. Each test returns P-value and, optionally, the test statistic of observed data, test statistic for all permutations, and other optional outputs. Some functions produce graph plots.

Many methodologies for testing the differential expression of gene sets have been suggested and are collectively named gene set analysis (GSA). GSA can be either *competitive* or *self-contained*. Competitive approaches compare a gene set against its complement which contains all genes excluding the genes in the set, and self-contained approaches compare whether a gene set is differentially expressed (DE) between two phenotypes (condition). Some competitive approaches are influenced by the genomic coverage and the filtering of the data and can increase their power by the addition of unrelated data and even noise [4] while others can be influenced by the proportion of up-regulated and down-regulated genes in a gene set between two conditions [5]. Due to these problems, package *GSAR* focuses on self-contained methods only. The possibility to formulate different statistical hypotheses by using different test statistics with self-contained approaches enables the formulation and exploration of different biological hypotheses [2]. For GSA, testing hypotheses other than the equality of the mean expression vectors remains underexplored. Package *GSAR* provides a set of methods to test a null hypothesis against specific alternatives, such as differential distribution (function `WWtest`), shift or mean (functions `KStest` and `MDtest`), scale or variance (functions `RKStest`, `RMDtest`, and `AggrFtest`) or net correlation structure (function `GSNCAtest`).

Most of the statistical methods available in package *GSAR* (all except `GSNCAtest` and `AggrFtest`) are network or graph-based. *GSAR* handles graphs using the rich functionality of the *igraph* class from package *igraph* [6]. *GSAR* invokes some functions from package *igraph* in its methods implementation and uses the plot method for class *igraph* for visualizing the generated graphs.

Data packages *ALL*, *GSVAdata* and *tweeDEseqCountData* which contain datasets are necessary for running the examples and case studies in this vignette. Package *GSAR* itself contains one preprocessed dataset to illustrate the analyses, which was employed in the article introducing the gene sets net correlations analysis (GSNCA) method [1]. Other packages necessary for running the examples and case studies in this vignette are packages *MASS*, *GSEABase*, *annotate*, *org.Hs.eg.db*, *genefilter*, *hgu95av2.db* and *edgeR*. The analysis will start by loading package *GSAR*

```
> library(GSAR)
```

In what follows we introduce the following notations. Consider two different biological phenotypes, with n_1 samples of measurements for the first and n_2 samples of the same measurements for the second. Each sample is a p-dimensional vector of measurements of p genes (constituting a single gene set). Hence, the data for the first phenotype consists of a $p \times n_1$ matrix and for the second phenotype consists of a $p \times n_2$ matrix, where rows are genes and columns are samples. The samples of the first and second phenotypes are respectively represented by two random vectors X and Y. Let X and Y be independent and identically distributed with the distribution functions F_x, F_y, p-dimensional mean vectors $\bar{X}$ and $\bar{Y}$, and $p \times p$ covariance matrices S_x and S_y.

2 Minimum spanning trees

2.1 First MST

The pooled multivariate (p-dimensional) observations X and Y can be represented by an edge-weighted graph $G(V, E)$ where V is the set of vertices in the graph. Each vertex in the sample network corresponds to one observation (sample) and E is the set of edges connecting pairs of vertices. The complete graph of X and Y has $N = n_1 + n_2$ vertices and $N(N-1)/2$ edges. The weights of the edges are estimated by the Euclidean distances between pairs of observations (samples) in R^p.

The minimum spanning tree (MST) is defined as the acyclic subset $T_1 \subseteq E$ that connects all vertices in V and whose total length $\sum_{i,j \in T_1} d(v_i, v_j)$ is minimal. Each vertex in the graph corresponds to a p-dimensional observation from X or Y. The MST provides a way of ranking the multivariate observations by giving them ranks according to the positions of their corresponding vertices in the MST. The purpose of this ranking is to obtain the strong relationship between observations differences in ranks and their distances in R^p. The ranking algorithm can be designed specifically to confine a particular alternative hypothesis more detection power [2]. Five tests in package *GSAR* are based on MST: `WWtest`, `KStest`, `MDtest`, `RKStest`, and `RMDtest`.

The following example generates a feature set of 20 features and 40 observations using the random multivariate normal data generator from package *MASS*, creates a graph object from the data and obtain its MST using functions from package *igraph*.

```
> library(MASS)
> set.seed(123)
> nf <- 20
```

```
> nobs <- 60
> zero_vector <- array(0,c(1,nf))
> cov_mtrx <- diag(nf)
> dataset <- mvrnorm(nobs, zero_vector, cov_mtrx)
> Wmat <- as.matrix(dist(dataset, method="euclidean", diag=TRUE,
+ upper=TRUE, p=2))
> gr <- graph_from_adjacency_matrix(Wmat, weighted=TRUE, mode="undirected")
> MST <- mst(gr)
```

2.2 MST2 for correlation and PPI networks

The second MST is defined as the MST of the reduced graph $G(V, E - T_1)$. We denote the union of the first and second MSTs by MST2. Each vertex in the MST2 has a minimum degree of 2 if all the edges between vertices are considered (full network).

The correlation (coexpression) network is defined as the edge-weighted graph $G(V, E)$ where V is the set of vertices in the graph with each vertex corresponding to one feature (gene) in the gene set and E is the set of edges connecting pairs of vertices with weights estimated by some correlation distance measure. The correlation distance here is defined by $d_{ij} = 1 - |r_{ij}|$ where d_{ij} and r_{ij} are respectively the correlation distance and correlation coefficient between genes i and j [1]. The MST2 of the correlation network gives the minimal set of essential links (interactions) among genes, which we interpret as a network of functional interactions. A gene that is highly correlated with most of the other genes in the gene set tends to occupy a central position and has a relatively high degree in the MST2 because the shortest paths connecting the vertices of the first and second MSTs tend to pass through this gene. In contrast, a gene with low intergene correlations most likely occupies a non-central position in the MST2 and has a degree of 2. This property of the MST2 makes it a valuable graphical visualization tool to examine the full correlation network obtained from gene expression data by highlighting the most influential genes. As an example, the MST2 of the dataset generated in the previous example can be found as follows

```
> ## The input of findMST2 must be a matrix with rows and columns
> ## respectively corresponding to genes and columns.
> ## Therefore, dataset must be transposed first.
> dataset <- aperm(dataset, c(2,1))
> MST2 <- findMST2(dataset)
```

The protein-protein Interaction (PPI) network is defined as the binary graph $G(V, E)$ where V is the set of vertices in the graph with each vertex corresponding to one protein and E is the set of edges connecting pairs of vertices where $e_{ij} = 1$ if interaction exists between proteins i and j and $e_{ij} = 0$ otherwise. The MST2 of the PPI network gives the minimal set of essential interactions among proteins. It was shown in [7] that function `findMST2.PPI` reveals fine network structure with highly connected proteins occupying central positions in clusters.

3 Statistical methods

Package *GSAR* provides seven statistical methods that test five specific alternative hypotheses against the null (see Figure 1), two functions to find the MST2 of correlation and PPI networks, one wrapper function to plot the MST2 of correlation networks obtained from gene expression data under two conditions, and one wrapper function to facilitate performing a specific statistical method for a list of gene sets.

3.1 Wald-Wolfowitz test

The Wald-Wolfowitz (WW) tests the null hypothesis $H_0 : F_x = F_y$ against the alternative $H_1 : F_x \neq F_y$. When $p = 1$, the univariate WW test begins by sorting the observations from two phenotypes in ascending order and labeling each observation by its phenotype. Then, the number of *runs* (R) is calculated where R is a consecutive sequence of identical labels. The test statistic is a function of the number of runs and is asymptotically normally distributed.

The multivariate generalization $(p > 1)$ suggested in [3] is based on the MST. Similar to the univariate case, in the multivariate generalization of WW test, all edges in the MST connecting two vertices (observations) with different

labels are removed and the number of the remaining disjoint trees (R) is calculated [3]. The test statistic is the standardized number of subtrees

$$W = \frac{R - E[R]}{\sqrt{var[R]}}$$

The null distribution of W is obtained by permuting the observation labels for a large number of times ($nperm$) and calculating W for each time. The null distribution is asymptotically normal. P-value is calculated as

$$P - value = \frac{\sum_{k=1}^{nperm} I\left[W_k \leq W_{obs}\right] + 1}{nperm + 1}$$

where W_k is the test statistic for permutation k, W_{obs} is the observed test statistic, and I is the indicator function. Function `WWtest` performs this test.

3.2 Kolmogorov-Smirnov tests

When $p = 1$, the univariate Kolmogorov-Smirnov (KS) test begins by sorting the observations from two phenotypes in ascending order. Then observations are ranked and the quantity

$$d_i = \frac{r_i}{n_1} - \frac{s_i}{n_2}$$

is calculated where r_i (s_i) is the number of observations in X (Y) ranked lower than i, $1 \leq i \leq N$. The test statistic is the maximal absolute difference between the Cumulative Distribution Functions (CDFs) of the ranks of samples from X and Y, $D = \sqrt{\frac{n_1 n_2}{n_1+n_2}} max|d_i|$. The null distribution of D is obtained by permuting the observation labels for a large number of times ($nperm$) and calculating D for each time. The KS statistic asymptotically follows the Smirnov distribution and tests a one-sided hypothesis. P-value is calculated as

$$P - value = \frac{\sum_{k=1}^{nperm} I\left[D_k \geq D_{obs}\right] + 1}{nperm + 1}$$

where D_k is the test statistic for permutation k, D_{obs} is the observed test statistic, and I is the indicator function.

The ranking scheme can be designed to confine a specific alternative hypothesis more power. Two possibilities are available: First, if the null hypothesis $H_0 : \bar{\mu_X} = \bar{\mu_Y}$ is tested against the alternative $H_1 : \bar{\mu_X} \neq \bar{\mu_Y}$, the MST is rooted at a node with the largest geodesic distance and the rest of the nodes are ranked according to the *high directed preorder* (HDP) traversal of the tree [3], which can be found using function `HDP.ranking`. Function `KStest` performs this specific test. Second, if the null hypothesis $H_0 : \bar{\sigma_X} = \bar{\sigma_Y}$ is tested against the alternative hypothesis $H_1 : \bar{\sigma_X} \neq \bar{\sigma_Y}$, the MST is rooted at the node of smallest geodesic distance (centroid) and nodes with largest depths from the root are assigned higher ranks. Hence, ranks are increasing *radially* from the root of the MST. The radial ranking of vertices in a tree can be found using function `radial.ranking`. Function `RKStest` performs this specific test.

The MST found in the previous example is shown in Figure 2 were vertices from group 1 are in green color and vertices from group 2 are in yellow color. Ranking vertices in the graph according to the HDP and radial traversal of the MST can be done respectively using functions `HDP.ranking` and `radial.ranking`

```
> HDP.ranking(MST)

 [1] 29 19 16 54 44 28 34 20 60 15 42 17  7 23 27 25 48 40  4 41 12  8 32 22 43
[26] 10 24  6 47 31 18 56 37 35 39 55 57 50  9  3 33 51 49  5 11 59 14 53  1 46
[51] 58 45 38 13 26 52 30 21  2 36

> radial.ranking(MST)

 [1]  6 55 24 47 42  3 57 31 18 37 56 17 34 33 49 40 50  9 35 23 25 41  4 52 58
[26]  7 59 60 20 28  5 51 39 27  8 45 48 14  1 54 12 30 21 15 11 22 53 44 38 32
[51] 46 19  2 43 13 10 26 16 29 36
```

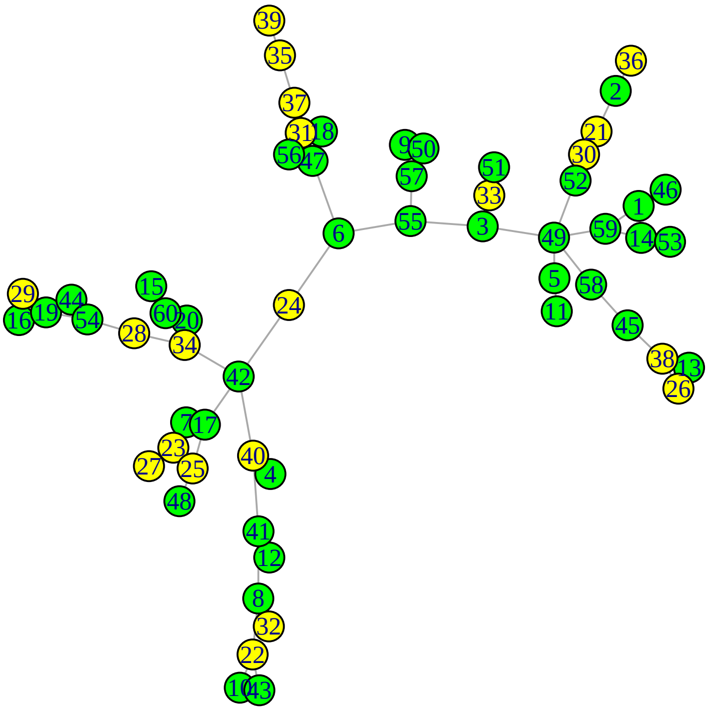

Figure 2: Minimum spanning tree of some random data.

3.3 Mean deviation tests

The mean deviation (MD) statistic calculates the average deviation between the Cumulative Distribution Functions (CDFs) of the ranks of samples from X and Y. The MD statistic for a gene set of size p is defined as

$$D = \sum_{i=1}^{p} [P(X, i) - P(Y, i)]$$

where

$$P(X, i) = \frac{\sum_{j \in X, j \leq i} r_j^{\alpha}}{\sum_{j \in X} r_j^{\alpha}}$$

and

$$P(Y, i) = \sum_{j \in Y, j \leq i} \frac{1}{n_2}$$

r_j is the rank of sample j in the MST and the exponent α is set to 0.25 to give the ranks a modest weight. The null distribution of D is obtained by permuting sample labels for a large number of times ($nperm$) and calculating D for each time. The MD statistic has asymptotically a normal distribution and tests a two-sided hypothesis. P-value is calculated as

$$P - value = \frac{\sum_{k=1}^{nperm} I\left[|D_k| \geq |D_{obs}|\right] + 1}{nperm + 1}$$

where D_k is the test statistic for permutation k, D_{obs} is the observed test statistic, and I is the indicator function. Similar to KS statistic, combining this statistic with appropriate ranking schemes, allows the test of specific alternative hypotheses, namely diffferential mean (mean deviation test function `MDtest`) and differential variance (radial mean deviation function `RMDtest`).

3.4 Aggregated F-test

The univariate F-test is used to detect differential variance in individual genes forming a gene set of size p, and the individual P-values, $p_i, 1 \leq i \leq p$, are then aggregated using Fisher probability combining method to obtain a score statistic (T) for the gene set

$$T = -2\sum_{i=1}^{p} \log_e(p_i)$$

Significance is estimated by permuting sample labels and calculating T for a large number of times ($nperm$). P-value is calculated as

$$P - value = \frac{\sum_{k=1}^{nperm} I\left[T_k \geq T_{obs}\right] + 1}{nperm + 1}$$

where T_k is the test statistic for permutation k, T_{obs} is the observed test statistic, and I is the indicator function.

3.5 Gene sets net correlations analysis

3.5.1 Method

Gene sets net correlations analysis (GSNCA) is a two-sample nonparametric multivariate differential coexpression test that accounts for the correlation structure between features (genes). For a gene set of size p, the test assigns weight factors $w_i, 1 \leq i \leq p$, to genes under one condition and adjust these weights simultaneously such that equality is achieved between each genes's weight and the sum of its weighted absolute correlations (r_{ij}) with other genes in a gene set of p genes

$$w_i = \sum_{j \neq i} w_i \left|r_{ij}\right| \qquad 1 \leq i \leq p$$

The problem is solved as an eigenvector problem with a unique solution which is the eigenvector corresponding to the largest eigenvalue of the genes' correlation matrix (see [1] for details).

The test statistic w_{GSNCA} is given by the first norm between the scaled weight vectors $w^{(1)}$ and $w^{(2)}$ (each vector is multiplied by its norm) between two conditions

$$w_{GSNCA} = \sum_{i=1}^{p} \left| w_i^{(1)} - w_i^{(2)} \right|$$

This test statistic tests the null hypothesis $H_0 : w_{GSNCA} = 0$ against the alternative $H_1 : w_{GSNCA} \neq 0$. The performance of this test was thoroughly examind in [1]. P-value is calculated in exactly the same way as before for the WW and KS tests. The values in the scaled weight vectors $w^{(1)}$ and $w^{(2)}$ roughly fall in the range $[0.5, 1.5]$, with high values indicating genes that are highly correlated with other genes in the same gene set.

3.5.2 The problem of zero standard deviation

In special cases some features in a set may have constant or nearly constant levels across the samples in one or both conditions. Such situation almost never encountered in microarray data, but may arises for RNA-seq count data where a gene may have zero counts under many samples if the gene is not expressed. This results in a zero or a tiny standard deviation. Such case produces an error in command `cor` used to compute the correlations between features. To avoid this situation, standard deviations are checked in advance (default behaviour) and if any is found below a specified minimum limit (default is `1e-3`), the execution stops and an error message is returned indicating the the number of feature causing the problem (if only one the index of that feature is given too). To perform the GSNCA for count data, the features causing the problem must be excluded from the set.

If a feature has nearly a constant level for some (but not all) samples under both conditions, permuting sample labels may group together such samples under one condition by chance and hence produce a standard deviation smaller than the minimum limit. To allow the test to skip such permutations without causing excessive delay, an upper limit for the number of allowed skips can be set (default is 10). If the upper limit is exceeded, an error message is returned.

If the user is certain that the tested feature sets contain no feature with nearly zero standard deviation (such as the case with filtered microarray data), the checking step for tiny standard deviations can be skipped in order to reduce the execution time.

4 Notes on handling RNA-seq count data

RNA-seq data consists of integer counts usually represented by the discrete Poisson or Negative Binomial distributions. Therefore, tests designed for microarray data (which follows the continuous normal distribution) can not be applied directly to RNA-seq data. The nonparametric tests presented in package *GSAR* need no prior distributional assumptions and can be applied to RNA-seq counts given that proper normalization is used. The normalization should accounts for the between-samples differences (library size or sequencing depth) and within-sample differences (mainly gene length). The *reads per kilobase per million* (RPKM) is such normalization. However, due to the lack of thorough performance studies, two points must be declared:

- The variance of both the Poisson and negative Bionomial distributions, used to model RNA-seq count data is a function of their mean. Therefore, applying the variance tests to RNA-seq data highly depends on the normalization method. This specific topic is not well-studied and characterized yet.
- RNA-seq datasets often have many zero counts, therefore, the problem of having at least one gene with zero standard deviations in a gene set is frequent and prevent calculating the correlation coefficients necessary to perform the GSNCA. One possible solution is to discard any genes that may have zero or tiny standard deviation and apply GSNCA to the remaining genes in the gene set.

5 Case studies

This Section illustrates the typical procedure for applying the methods available in package *GSAR* to perform GSA. Two microarray and one RNA-seq datasets are used.

5.1 The p53 dataset

5.1.1 Introduction

p53 is a major tumor suppressor protein. The p53 dataset comprises 50 samples of the NCI-60 cell lines differentiated based on the status of the TP53 gene: 17 cell lines carrying wild type (WT) TP53 and 33 cell lines carrying mutated (MUT) TP53 [8, 9]. Transcriptional profiles obtained from microarrays of platform hgu95av2 are available from the Broad Institute's website (`http://www.broadinstitute.org/gsea/datasets.jsp`).

5.1.2 Filtering and normalization

A preprocessed version of p53 dataset is available in package *GSAR* as a *matrix* object. The p53 dataset was dowloaded from the Broad Institute's website. Probe level intensities were quantile normalized and transformed to the log scale using $\log_2(1 + intensity)$. Probes originally had Affymetrix identifiers which are mapped to unique gene symbol identifiers. Probes without mapping to entrez and gene symbol identifiers were discarded. Probes with duplicate intensities were assessed and the probe with the largest absolute value of t-statistic between WT and MUT conditions was selected as the gene match. Finally, genes were assigned gene symbol identifiers and columns were assigned names indicating weither they belong to WT or MUT group. The columns were sorted such that the first 17 columns are WT samples and the next 33 columns are the MUT samples. This processed version of the p53 dataset was used in the analysis presented in [1].

5.1.3 GSA

GSA is performed on selected C2 curated gene sets (pathways) of the *molecular signatures database* (MSigDB) 3.0 [10]. This list of gene sets is available in package *GSVAdata*. We start by loading the required data

```
> library(GSVAdata)
> data(p53DataSet)
> data(c2BroadSets)
```

`c2BroadSets` is an object of class *GeneSetCollection* supported by package *GSEABase*. The genes in the `c2BroadSets` object have entrez identifiers. Package *org.Hs.eg.db* is used to convert the entrez identifiers to gene symbol identifiers. Genes without unique mapping to gene symbol identifiers or that do not exist in the p53 dataset are discarded from the C2 pathways. This insures proper indexing of genes in the dataset by the gene names in each C2 pathway. Finally, we keep only pathways with $10 \leq p \leq 500$ where p is the number of genes remaining in the pathways after filtering steps.

```
> library(org.Hs.eg.db)
> library(GSEABase)
> C2 <- as.list(geneIds(c2BroadSets))
> len <- length(C2)
> genes.entrez <- unique(unlist(C2))
> genes.symbol <- array("",c(length(genes.entrez),1))
> x <- org.Hs.egSYMBOL
> mapped_genes <- mappedkeys(x)
> xx <- as.list(x[mapped_genes])
> for (ind in 1:length(genes.entrez)){
+     if (length(xx[[genes.entrez[ind]]])!=0)
+         genes.symbol[ind] <- xx[[genes.entrez[ind]]]
+                                     }
> ## discard genes with no mapping to gene symbol identifiers
> genes.no.mapping <- which(genes.symbol == "")
> if(length(genes.no.mapping) > 0){
+     genes.entrez <- genes.entrez[-genes.no.mapping]
+     genes.symbol <- genes.symbol[-genes.no.mapping]
+                                 }
> names(genes.symbol) <- genes.entrez
> ## discard genes in C2 pathways which do not exist in p53 dataset
> p53genes <- rownames(p53DataSet)
> remained <- array(0,c(1,len))
> for (k in seq(1, len, by=1)) {
+     remained[k] <- sum((genes.symbol[C2[[k]]] %in% p53genes) &
+     (C2[[k]] %in% genes.entrez))
+                             }
> ## discard C2 pathways which have less than 10 or more than 500 genes
> C2 <- C2[(remained>=10)&&(remained<=500)]
> pathway.names <- names(C2)
```

```
> c2.pathways <- list()
> for (k in seq(1, length(C2), by=1)) {
+     selected.genes <- which(p53genes %in% genes.symbol[C2[[k]]])
+     c2.pathways[[length(c2.pathways)+1]] <- p53genes[selected.genes]
+                                  }
> names(c2.pathways) <- pathway.names
> path.index <- which(names(c2.pathways) == "LU_TUMOR_VASCULATURE_UP")
```

c2.pathways is now a list with each entry being a named list of the genes (gene symbol identifiers) forming one C2 pathway. To demonstrate the use of different tests, we consider the C2 pathway LU TUMOR VASCULATURE UP used in [1] to illustrate GSNCA.

```
> target.pathway <- p53DataSet[c2.pathways[["LU_TUMOR_VASCULATURE_UP"]],]
> group.label <- c(rep(1,17), rep(2,33))
> WW_pvalue <- WWtest(target.pathway, group.label)
> KS_pvalue <- KStest(target.pathway, group.label)
> MD_pvalue <- MDtest(target.pathway, group.label)
> RKS_pvalue <- RKStest(target.pathway, group.label)
> RMD_pvalue <- RMDtest(target.pathway, group.label)
> F_pvalue <- AggrFtest(target.pathway, group.label)
> GSNCA_pvalue <- GSNCAtest(target.pathway, group.label)
> WW_pvalue
```

```
[1] 0.125
```

```
> KS_pvalue
```

```
[1] 0.0529
```

```
> MD_pvalue
```

```
[1] 0.049
```

```
> RKS_pvalue
```

```
[1] 0.101
```

```
> RMD_pvalue
```

```
[1] 0.205
```

```
> F_pvalue
```

```
[1] 0.869
```

```
> GSNCA_pvalue
```

```
[1] 0.029
```

The questions addressed by these tests were the identification of gene sets expressed with different distributions, means, variances or correlation structure between two conditions. At a significance level 0.05, the targeted pathway shows a statistical evidence of being differentially coexpressed only. The MST2s of the correlation network for WT and MUT groups are shown in Figure 3, generated by function plotMST2.pathway

```
> plotMST2.pathway(object=p53DataSet[c2.pathways[[path.index]],],
+ group=c(rep(1,17), rep(2,33)), name="LU_TUMOR_VASCULATURE_UP",
+ legend.size=1.2, leg.x=-1.2, leg.y=2,
+ label.size=1, label.dist=0.8, cor.method="pearson")
```

The targeted pathway comprises genes over-expressed in ovarian cancer endothelium [11]. Gene TNFAIP6 (tumor necrosis factor, α-induced protein 6) identified by GSNCA as a hub gene for WT group and visualized using MST2 (Figure 3, left panel) was found 29.1-fold over-expressed in tumor endothelium in the original study and was suggested to be specific for ovarian cancer vasculature. This indicates that gene TNFAIP6 can be an important regulator of ovarian cancer, and identifying it as a hub by GSNCA enhances the original observation. When p53 is mutated (Figure 3, right panel) the hub gene is VCAN, containing p53 binding site and its expression is highly correlated with p53 dosage [12]. Therefore, both hub genes provide adequate information about the underlying biological processes.

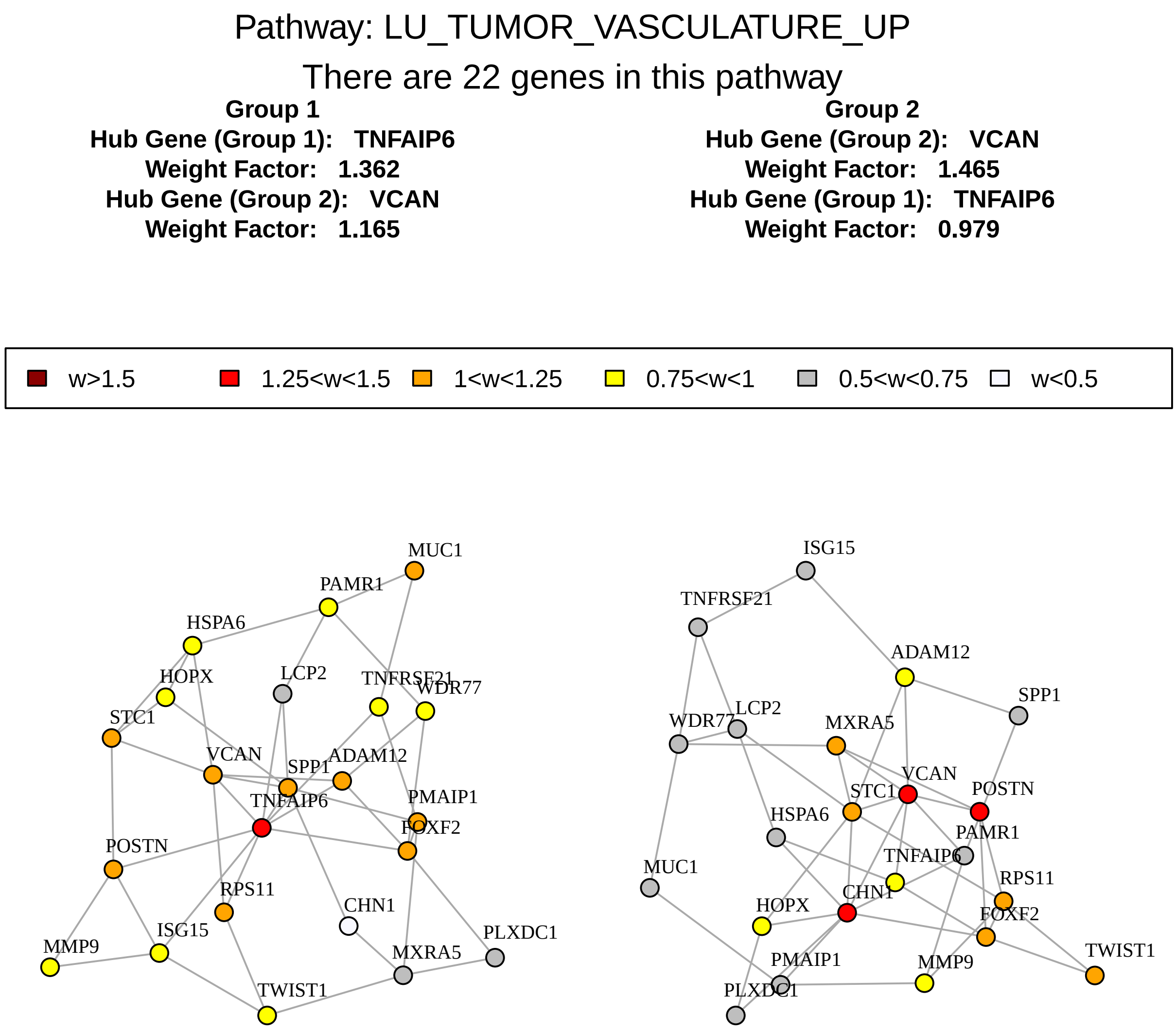

Figure 3: MST2s of LU TUMOR VASCULATOR UP correlation network, (left) WT (right) MUT.

If testing multiple or all the gene sets in the `c2.pathways` list is desired, the wrapper function `TestGeneSets` can be used. For example, the following code tests the first 3 gene sets in list `c2.pathways`, that have a minimum of 10 and a maximum of 100 genes, using the GSNCA method

```
> results <- TestGeneSets(object=p53DataSet, group=group.label,
+ geneSets=c2.pathways[1:3], min.size=10, max.size=100, test="GSNCAtest")
> results
```

```
$NAKAMURA_CANCER_MICROENVIRONMENT_UP
[1] 0.722

$NAKAMURA_CANCER_MICROENVIRONMENT_DN
[1] 0.801

$WEST_ADRENOCORTICAL_TUMOR_MARKERS_UP
```

```
[1] 0.656
```

5.2 The ALL dataset

5.2.1 Introduction

This dataset consists of microarrays (platform hgu95av2) from 128 different individuals with acute lymphoblastic leukemia (ALL). There are 95 samples with B-cell ALL [13] and 33 with T-cell ALL [14]. We consider B-cell type only and compare tumors carrying the BCR/ABL mutations (37 samples) to those with no cytogenetic abnormalities (42 samples). The Bioconductor package *ALL* provides the ALL dataset with samples normalized using the *robust multiarray analysis* (RMA) procedure [15].

5.2.2 Filtering and normalization

Affymetrix probe identifiers without mapping to entrez and gene symbol identifiers were discarded. Affymetrix probe identifiers were mapped to unique gene symbol identifiers and intensities of probes mapping to the same gene symbol identifier were assessed and the probe with the largest absolute value of t-statistic between normal (NEG) and mutation (MUT) conditions was selected as the gene match.

```
> library(Biobase)
> library(genefilter)
> library(annotate)
> library(hgu95av2.db)
> library(ALL)
> data(ALL)
> bcell = grep("^B", as.character(ALL$BT))
> types = c("NEG", "BCR/ABL")
> moltyp = which(as.character(ALL$mol.biol) %in% types)
> ALL_bcrneg = ALL[, intersect(bcell, moltyp)]
> ALL_bcrneg$mol.biol = factor(ALL_bcrneg$mol.biol)
> ALL_bcrneg$BT = factor(ALL_bcrneg$BT)
> nBCR <- sum(ALL_bcrneg$mol.biol == "BCR/ABL")
> nNEG <- sum(ALL_bcrneg$mol.biol == "NEG")
> BCRsamples <- which(ALL_bcrneg$mol.biol == "BCR/ABL")
> NEGsamples <- which(ALL_bcrneg$mol.biol == "NEG")
> ALL_bcrneg <- ALL_bcrneg[,c(BCRsamples,NEGsamples)]
> platform <- annotation(ALL_bcrneg)
> annType <- c("db", "env")
> entrezMap <- getAnnMap("ENTREZID", annotation(ALL_bcrneg),
+ type=annType, load=TRUE)
> symbolMap <- getAnnMap("SYMBOL", annotation(ALL_bcrneg),
+ type=annType, load=TRUE)
> filtered <- nsFilter(ALL_bcrneg, require.entrez=TRUE,
+ remove.dupEntrez=FALSE, require.symbol=TRUE, require.GOBP=FALSE,
+ var.func=IQR, var.filter=FALSE, var.cutof=0.5)
> filtered.set <- filtered$eset
> probe.names <- featureNames(filtered.set)
> rr <- rowttests(filtered.set, as.factor(ALL_bcrneg$mol.biol), tstatOnly=TRUE)
> fL <- findLargest(probe.names, abs(rr$statistic), platform)
> filtset2 <- filtered.set[fL,]
> affymetrix.probe.names <- featureNames(filtset2)
> gene.symbols <- lookUp(affymetrix.probe.names, platform, "SYMBOL")
> featureNames(filtset2) <- gene.symbols
> ALLdataset <- exprs(filtset2)
```

5.2.3 Selected gene set

Lets examine the C2 pathway KEGG CHRONIC MYELOID LEUKEMIA, knowm to be specifically associated with the BCR/ABL mutation. This pathway has many BCR/ABL-related genes and hence expected to show difference between NEG and MUT conditions. To ensure proper indexing, the lists of genes in C2 pathways should consists only of genes available in the filtered ALL dataset. Therefore, the same steps taken to filter the C2 pathways with the p53 dataset are repeated for the ALL dataset.

```
> C2 <- as.list(geneIds(c2BroadSets))
> len <- length(C2)
> genes.entrez <- unique(unlist(C2))
> genes.symbol <- array("",c(length(genes.entrez),1))
> x <- org.Hs.egSYMBOL
> mapped_genes <- mappedkeys(x)
> xx <- as.list(x[mapped_genes])
> for (ind in 1:length(genes.entrez)){
+     if (length(xx[[genes.entrez[ind]]])!=0)
+         genes.symbol[ind] <- xx[[genes.entrez[ind]]]
+                                     }
> ## discard genes with no mapping to gene symbol identifiers
> genes.no.mapping <- which(genes.symbol == "")
> if(length(genes.no.mapping) > 0){
+     genes.entrez <- genes.entrez[-genes.no.mapping]
+     genes.symbol <- genes.symbol[-genes.no.mapping]
+                                  }
> names(genes.symbol) <- genes.entrez
> ## discard genes in C2 pathways which do not exist in ALL dataset
> ALLgenes <- rownames(ALLdataset)
> remained <- array(0,c(1,len))
> for (k in seq(1, len, by=1)) {
+     remained[k] <- sum((genes.symbol[C2[[k]]] %in% ALLgenes) &
+     (C2[[k]] %in% genes.entrez))
+                               }
> ## discard C2 pathways which have less than 10 or more than 500 genes
> C2 <- C2[(remained>=10)&&(remained<=500)]
> pathway.names <- names(C2)
> c2.pathways <- list()
> for (k in seq(1, length(C2), by=1)) {
+     selected.genes <- which(ALLgenes %in% genes.symbol[C2[[k]]])
+     c2.pathways[[length(c2.pathways)+1]] <- ALLgenes[selected.genes]
+                                     }
> names(c2.pathways) <- pathway.names
> path.index <- which(names(c2.pathways) == "KEGG_CHRONIC_MYELOID_LEUKEMIA")
```

c2.pathways is now a list with each entry being a named list of the genes (gene symbol identifiers) forming one C2 pathway. Only genes available in the filtered ALL dataset are included in the pathways.

```
> KCMLpathway <- ALLdataset[c2.pathways[["KEGG_CHRONIC_MYELOID_LEUKEMIA"]],]
> group.label <- c(rep(1,37), rep(2,42))
> WW_pvalue <- WWtest(KCMLpathway, group.label)
> KS_pvalue <- KStest(KCMLpathway, group.label)
> MD_pvalue <- MDtest(KCMLpathway, group.label)
> RKS_pvalue <- RKStest(KCMLpathway, group.label)
> RMD_pvalue <- RMDtest(KCMLpathway, group.label)
> F_pvalue <- AggrFtest(KCMLpathway, group.label)
> GSNCA_pvalue <- GSNCAtest(KCMLpathway, group.label)
> WW_pvalue
```

```
[1] 0.005
```

```
> KS_pvalue
```

```
[1] 0.733
> MD_pvalue
[1] 0.695
> RKS_pvalue
[1] 0.414
> RMD_pvalue
[1] 0.293
> F_pvalue
[1] 0.0669
> GSNCA_pvalue
[1] 0.005
```

At a significance level 0.05, P-values show statistical evidence that the pathway is differentially coexpressed and has different distribution between BCR/ABL and NEG conditions. The MST2s of the correlation network for BCR/ABL and NEG groups are shown in Figure 4, generated by function `plotMST2.pathway`

```
> plotMST2.pathway(object=KCMLpathway, group=group.label,
+ name="KEGG_CHRONIC_MYELOID_LEUKEMIA", legend.size=1.2, leg.x=-1,
+ leg.y=2, label.size=0.8, cor.method="pearson")
```

5.3 The Pickrell dataset

5.3.1 Introduction

The Pickrell dataset of sequenced cDNA libraries generated from 69 lymphoblastoid cell lines derived from unrelated Yoruban Nigerian individuals (YRI) is part of the HapMap project. The original experimental data was published by [16]. Package *tweeDEseqCountData* provides the table of counts for this dataset in the expression set object `pickrell.eset`. This table of counts corresponds to the one in the ReCount repository available at `http://bowtie-bio.sourceforge.net/recount`. Details on the pre-processing steps to obtain this table of counts from the raw reads are provided by [17].

Package *tweeDEseqCountData* provides annotation data for the human genes forming the table in `pickrell.eset` as a data frame object `annotEnsembl63`. *tweeDEseqCountData* also provides two lists of genes (gene sets) with documented sex-specific expression and occurring within the set of genes that form the table of counts in `pickrell.eset`. The first is a set of genes that are located on the male-specific region of chromosome Y, and therefore are over-expressed in males (`msYgenes`). The second is a set of genes, that are escaping X-chromosome inactivation, and therefore are overexpressed in females (`XiEgenes`). These two sets are useful in serving as true positives when GSA is conducted between males and females to detect gene sets that are differentially expressed.

```
> library(tweeDEseqCountData)
> data(pickrell)
> data(annotEnsembl63)
> data(genderGenes)
> gender <- pickrell.eset$gender
> pickrell.eset
```

```
ExpressionSet (storageMode: lockedEnvironment)
assayData: 52580 features, 69 samples
  element names: exprs
protocolData: none
phenoData
  sampleNames: NA18486 NA18498 ... NA19257 (69 total)
  varLabels: num.tech.reps population study gender
  varMetadata: labelDescription
featureData
```

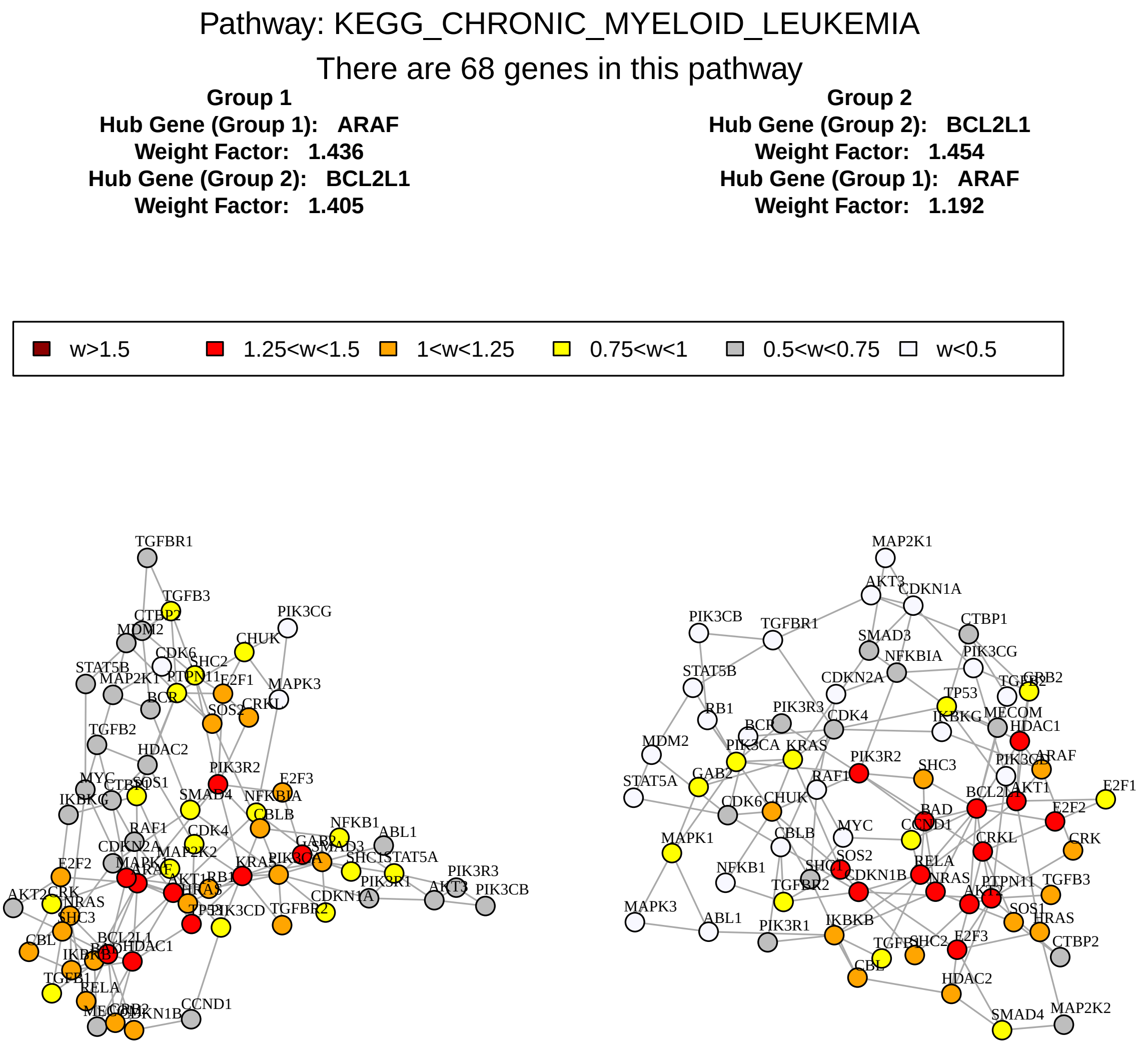

Figure 4: MST2s of pathway KEGG CHRONIC MYELOID LEUKEMIA correlation network, (left) BCR/ABL (right) NEG.

```
  featureNames: ENSG00000000003 ENSG00000000005 ... LRG_99 (52580
    total)
  fvarLabels: gene
  fvarMetadata: labelDescription
experimentData: use 'experimentData(object)'
Annotation:
```

```
> sampleNames(pickrell.eset)[gender == "male"]
```

```
 [1] "NA18486" "NA18498" "NA18501" "NA18504" "NA18507" "NA18510" "NA18516"
 [8] "NA18519" "NA18522" "NA18853" "NA18856" "NA18862" "NA18871" "NA18913"
[15] "NA19098" "NA19101" "NA19119" "NA19128" "NA19130" "NA19138" "NA19144"
[22] "NA19153" "NA19160" "NA19171" "NA19192" "NA19200" "NA19203" "NA19210"
[29] "NA19239"
```

```
> sampleNames(pickrell.eset)[gender == "female"]

 [1] "NA18499" "NA18502" "NA18505" "NA18508" "NA18511" "NA18517" "NA18520"
 [8] "NA18523" "NA18852" "NA18855" "NA18858" "NA18861" "NA18870" "NA18909"
[15] "NA18912" "NA18916" "NA19093" "NA19099" "NA19102" "NA19108" "NA19114"
[22] "NA19116" "NA19127" "NA19131" "NA19137" "NA19140" "NA19143" "NA19147"
[29] "NA19152" "NA19159" "NA19172" "NA19190" "NA19193" "NA19201" "NA19204"
[36] "NA19209" "NA19222" "NA19225" "NA19238" "NA19257"

> head(annotEnsembl63)

                Symbol Chr     Start       End EntrezID
ENSG00000252775     U7   5 133913821 133913880     <NA>
ENSG00000207459     U6   5 133970529 133970635     <NA>
ENSG00000252899     U7   5 133997420 133997479     <NA>
ENSG00000201298     U6   5 134036862 134036968     <NA>
ENSG00000222266     U6   5 134051173 134051272     <NA>
ENSG00000222924     U6   5 137405044 137405147     <NA>
                                                      Description Length
ENSG00000252775 U7 small nuclear RNA  [Source:RFAM;Acc:RF00066]     NA
ENSG00000207459  U6 spliceosomal RNA  [Source:RFAM;Acc:RF00026]     NA
ENSG00000252899 U7 small nuclear RNA  [Source:RFAM;Acc:RF00066]     NA
ENSG00000201298  U6 spliceosomal RNA  [Source:RFAM;Acc:RF00026]     NA
ENSG00000222266  U6 spliceosomal RNA  [Source:RFAM;Acc:RF00026]     NA
ENSG00000222924  U6 spliceosomal RNA  [Source:RFAM;Acc:RF00026]     NA
                GCcontent
ENSG00000252775        NA
ENSG00000207459        NA
ENSG00000252899        NA
ENSG00000201298        NA
ENSG00000222266        NA
ENSG00000222924        NA

> length(msYgenes)

[1] 32

> length(XiEgenes)

[1] 63
```

We will also extract the set of all X-linked genes that are not escaping inactivation (`Xigenes`) to use it as a true negative set (not differentially expressed)

```
> allXgenes <- rownames(annotEnsembl63)[annotEnsembl63$Chr == "X"]
> Xigenes <- allXgenes[!(allXgenes %in% XiEgenes)]
> length(Xigenes)

[1] 2249
```

5.3.2 Filtering and normalization

Any transcript without entrez identifier or gene length information is discarded. To consider only expressed genes in the analysis, genes with an average *count per million* (cpm) less than 0.1 are discarded. The gene length information is used to perform the RPKM normalization. Finally, the RPKM-normalized expression is transformed to the logarithm scale. RPKM as well as a few other normalizations were used with the Pickrell datasets in [18] to perform GSA and the study found no significant differences between different normalizations for the same test statistic.

```
> library(edgeR)
> gene.indices <- which(!(is.na(annotEnsembl63$EntrezID) |
+ is.na(annotEnsembl63$Length)))
> PickrellDataSet <- exprs(pickrell.eset)
```

```
> PickrellDataSet <- PickrellDataSet[gene.indices,]
> genes.length <- annotEnsembl63$Length[gene.indices]
> cpm.matrix <- cpm(PickrellDataSet)
> cpm.means <- rowMeans(cpm.matrix)
> cpm.filter <- which(cpm.means > 0.1)
> PickrellDataSet <- PickrellDataSet[cpm.filter,]
> genes.length <- genes.length[cpm.filter]
> rpkm.set <- rpkm(PickrellDataSet, genes.length)
> rpkm.set <- log2(1 + rpkm.set)
```

5.3.3 Testing selected pathways

Any gene in msYgenes, XiEgenes, or Xigenes but not found in the filtered dataset is discarded. Then, the remaining gender-related genes in msYgenes and XiEgenes are combined into one gene set (XYgenes).

```
> gene.space <- rownames(rpkm.set)
> msYgenes <- msYgenes[msYgenes %in% gene.space]
> XiEgenes <- XiEgenes[XiEgenes %in% gene.space]
> Xigenes <- Xigenes[Xigenes %in% gene.space]
> XYgenes <- c(msYgenes, XiEgenes)
> length(XYgenes)
```

```
[1] 14
```

```
> length(Xigenes)
```

```
[1] 147
```

The gender-related gene set XYgenes was found differentially expressed with high significance

```
> XYpathway <- rpkm.set[XYgenes,]
> group.label.pickrell <- (gender == "male") + 1
> WW_pvalue <- WWtest(XYpathway, group.label.pickrell)
> KS_pvalue <- KStest(XYpathway, group.label.pickrell)
> WW_pvalue
```

```
[1] 0.000999
```

```
> KS_pvalue
```

```
[1] 0.000999
```

while gene set Xigenes showed no such evidence as expected

```
> Xipathway <- rpkm.set[Xigenes,]
> WW_pvalue <- WWtest(Xipathway, group.label.pickrell)
> KS_pvalue <- KStest(Xipathway, group.label.pickrell)
> WW_pvalue
```

```
[1] 0.585
```

```
> KS_pvalue
```

```
[1] 0.495
```

To apply the GSNCA, genes with tiny standard deviations must be filtered out first

```
> nrow(XYpathway)
```

```
[1] 14
```

```
> nrow(Xipathway)
```

```
[1] 147
```

```
> tiny.sd.XY.female <- which(apply(XYpathway[, group.label.pickrell == 1], 1, "sd") < 1e-3)
> tiny.sd.XY.male <- which(apply(XYpathway[, group.label.pickrell == 2], 1, "sd") < 1e-3)
```

```
> tiny.sd.Xi.female <- which(apply(Xipathway[, group.label.pickrell == 1], 1, "sd") < 1e-3)
> tiny.sd.Xi.male <- which(apply(Xipathway[, group.label.pickrell == 2], 1, "sd") < 1e-3)
> length(tiny.sd.XY.female)
```

```
[1] 2
```

```
> length(tiny.sd.XY.male)
```

```
[1] 0
```

```
> length(tiny.sd.Xi.female)
```

```
[1] 0
```

```
> length(tiny.sd.Xi.male)
```

```
[1] 0
```

```
> apply(XYpathway[, group.label.pickrell == 1], 1, "sd")
```

```
ENSG00000183878 ENSG00000154620 ENSG00000198692 ENSG00000157828 ENSG00000173674
          0.000           0.214           0.000           0.206           0.572
ENSG00000186310 ENSG00000169100 ENSG00000189037 ENSG00000215301 ENSG00000147003
          0.162           0.342           0.163           0.773           0.465
ENSG00000182287 ENSG00000169895 ENSG00000196459 ENSG00000169906
          0.502           0.446           0.322           0.143
```

```
> if(length(tiny.sd.XY.male) > 0) XYpathway <- XYpathway[-tiny.sd.XY.male,]
> if(length(tiny.sd.XY.female) > 0) XYpathway <- XYpathway[-tiny.sd.XY.female,]
> if(length(tiny.sd.Xi.male) > 0) Xipathway <- Xipathway[-tiny.sd.Xi.male,]
> if(length(tiny.sd.Xi.female) > 0) Xipathway <- Xipathway[-tiny.sd.Xi.female,]
> nrow(XYpathway)
```

```
[1] 12
```

```
> nrow(Xipathway)
```

```
[1] 147
```

Notice that two genes (ENSG00000183878 and ENSG00000198692) in `XYpathway` had zero standard deviations for female samples and were filtered out from `XYpathway`. These two genes are Y-liked genes and expected to have many zero counts for female samples. Although this filtering step increases the chances of success in performing GSNCA, the existence of many zero counts dispersed over many samples for one or more genes may still cause a problem when the sample permutation process groups many zero counts under one condition. The parameter `max.skip` in function `GSNCAtest` allows some tolerance by assigning the maximum number of skipped permutations allowed to avoid the few ones causing the problem. This solution may work or fail depending on the proportion of zero counts in the data. For example, assigning `max.skip` to 100 or more solved the problem for `XYpathway`, but it did not for `Xipathway`. We advise to perform gene filtering based on zero counts prior to trying the GSNCA for count data.

6 Session info

```
> sessionInfo()
```

```
R version 3.3.0 (2016-05-03)
Platform: x86_64-w64-mingw32/x64 (64-bit)
Running under: Windows 7 x64 (build 7601) Service Pack 1

locale:
[1] LC_COLLATE=C
[2] LC_CTYPE=English_United States.1252
[3] LC_MONETARY=English_United States.1252
[4] LC_NUMERIC=C
[5] LC_TIME=English_United States.1252
```

```
attached base packages:
[1] stats4    parallel  stats     graphics  grDevices utils     datasets
[8] methods   base

other attached packages:
 [1] edgeR_3.14.0              limma_3.28.21
 [3] tweeDEseqCountData_1.10.0 ALL_1.14.0
 [5] hgu95av2.db_3.2.3         genefilter_1.54.2
 [7] GSVAdata_1.8.0            hgu95a.db_3.2.3
 [9] org.Hs.eg.db_3.3.0        GSEABase_1.34.1
[11] graph_1.50.0              annotate_1.50.1
[13] XML_3.98-1.4              AnnotationDbi_1.34.4
[15] IRanges_2.6.1             S4Vectors_0.10.3
[17] Biobase_2.32.0            BiocGenerics_0.18.0
[19] MASS_7.3-45               GSAR_1.9.1
[21] igraph_1.0.1

loaded via a namespace (and not attached):
 [1] magrittr_1.5    splines_3.3.0   lattice_0.20-34 xtable_1.8-2
 [5] tools_3.3.0     grid_3.3.0      DBI_0.5-1       survival_2.40-1
 [9] Matrix_1.2-7.1  bitops_1.0-6    RCurl_1.95-4.8  RSQLite_1.0.0
```

References

[1] Yasir Rahmatallah, Frank Emmert-Streib, and Galina Glazko. Gene sets net correlations analysis (GSNCA): a multivariate differential coexpression test for gene sets. *Bioinformatics*, 30(3):360–368, 2014.

[2] Yasir Rahmatallah, Frank Emmert-Streib, and Galina Glazko. Gene set analysis for self-contained tests: complex null and specific alternative hypotheses. *Bioinformatics*, 28(23):3073–3080, 2012.

[3] Jerome Friedman and Lawrence Rafsky. Multivariate generalization of the Wald-Wolfowitz and Smirnov two-sample tests. *The Annals of Statistics*, 7:697–717, 1979.

[4] Shailesh Tripathi, Galina Glazko, and Frank Emmert-Streib. Ensuring the statistical soundness of competitive gene set approaches: gene filtering and genome-scale coverage are essential. *Nucleic Acids Res.*, 41:e82, 2013.

[5] Yasir Rahmatallah, Frank Emmert-Streib, and Galina Glazko. Gene set analysis approaches for RNA-seq data: performance evaluation and application guideline. *Briefings in Bioinformatics*, 17(3):393–407, 2016.

[6] Gabor Csardi and Tamas Nepusz. The igraph software package for complex network research. *InterJournal*, Complex Systems:1695, 2006. URL: http://igraph.org.

[7] Boris Zybailov, Alicia Byrd, Galina Glazko, Yasir Rahmatallah, and Kevin Raney. Protein-protein interaction analysis for functional characterization of helicases. *Methods*, 108(1):56–64, 2016.

[8] Magali Olivier, Ros Eeles, Monica Hollstein, Mohammed Khan, Curtis Harris, and Pierre Hainaut. The IARC TP53 database: new online mutation analysis and recommendations to users. *Human Mutation*, 19(6):607–614, 2002.

[9] Aravind Subramanian, Pablo Tamayo, Vamsi Mootha, Sayan Mukherjee, Benjamin Ebert, Michael Gillette, Amanda Paulovich, Scott Pomeroy, Todd Golub, Eric Lander, and Jill Mesirov. Gene set enrichment analysis: A knowledge-based approach for interpreting genome-wide expression profiles. *Proc. Natl. Acad. Sci.*, 102(43):15545–15550, 2005.

[10] Arthur Liberzon, Aravind Subramanian, Reid Pinchback, Helga Thorvaldsdottir, Pablo Tamayo, and Jill Mesirov. Molecular signatures database (MSigDB) 3.0. *Bioinformatics*, 27(12):1739–1740, 2011.

[11] Chunhua Lu, Tomas Bonome, Yang Li, Aparna Kamat, Liz Han, Rosemarie Schmandt, Robert Coleman, David Gershenson, Robert Jaffe, Michael Birrer, and Anil Sood. Gene alterations identified by expression profiling in tumor-associated endothelial cells from invasive ovarian carcinom. *Cancer Research*, 67(4):1757–1768, 2007.

[12] Heejei Yoon, Sandya Liyanarachchi, Fred Wright, Ramana Davuluri, Janet Lockman, Albert de la Chapelle, and Natalia Pellegata. Gene expression profiling of isogenic cells with different TP53 gene dosage reveals numerous genes that are affected by TP53 dosage and identifies CSPG2 as a direct target of p53. *Proceedings of the National Academy of Science*, 99(24):15632–15637, 2002.

[13] Sabina Chiaretti, Xiaochun Li, Robert Gentleman, Antonella Vitale, Marco Vignetti, Franco Mandelli, Jerome Ritz, and Robin Foa. Gene expression profile of adult T-cell acute lymphocytic leukemia identifies distinct subsets of patients with different response to therapy and survival. *Blood*, 103(7):2771–2778, 2004.

[14] Sabina Chiaretti, Xiaochun Li, Robert Gentleman, Antonella Vitale, Kathy Wang, Franco Mandelli, Robin Foa, and Jerome Ritz. Gene expression profiles of B-lineage adult acute lymphocytic leukemia reveal genetic patterns that identify lineage derivation and distinct mechanisms of transformation. *Clinical Cancer Research*, 11(20):7209–7219, 2005.

[15] Rafael Irizarry, Bridget Hobbs, Francois Collin, Yasmin Beazer-Barclay, Kristen Antonellis, Uwe Scherf, and terence Speed. Exploration, normalization, and summaries of high density oligonucleotide array probe level data. *Biostatistics*, 4(2):249–264, 2003.

[16] Joseph K. Pickrell, John C. Marioni, Athma A. Pai, Jacob F. Degner, Barbara E. Engelhardt, Everlyne Nkadori, Jean-Baptiste Veyrieras, Matthew Stephens, Yoav Gilad, and Jonathan K. Pritchard. Understanding mechanisms underlying human gene expression variation with RNA sequencing. *Nature*, 464:768–772, 2010.

[17] Alyssa C. Frazee, Ben Langmead, and Jeffrey T. Leek. ReCount: a multi-experiment resource of analysis-ready RNA-seq gene count datasets. *BMC Bioinformatics*, 12:449, 2011.

[18] Yasir Rahmatallah, Frank Emmert-Streib, and Galina Glazko. Comparative evaluation of gene set analysis approaches for RNA-Seq data. *BMC Bioinformatics*, 15:397, 2014.
