# Supplementary material for: GSAR: Bioconductor package for Gene Set analysis in R
Source: BMC Bioinformatics. 2017 Jan 24;18:61. doi: 10.1186/s12859-017-1482-6 (PMC5259853; doi:10.1186/s12859-017-1482-6)
Supplement: Additional file 3: — Source file ‘GSAR_1.9.1.tar.gz’. (GZ 2174 kb) [file 12859_2017_1482_MOESM3_ESM.gz › GSAR/vignettes/outline.pdf]

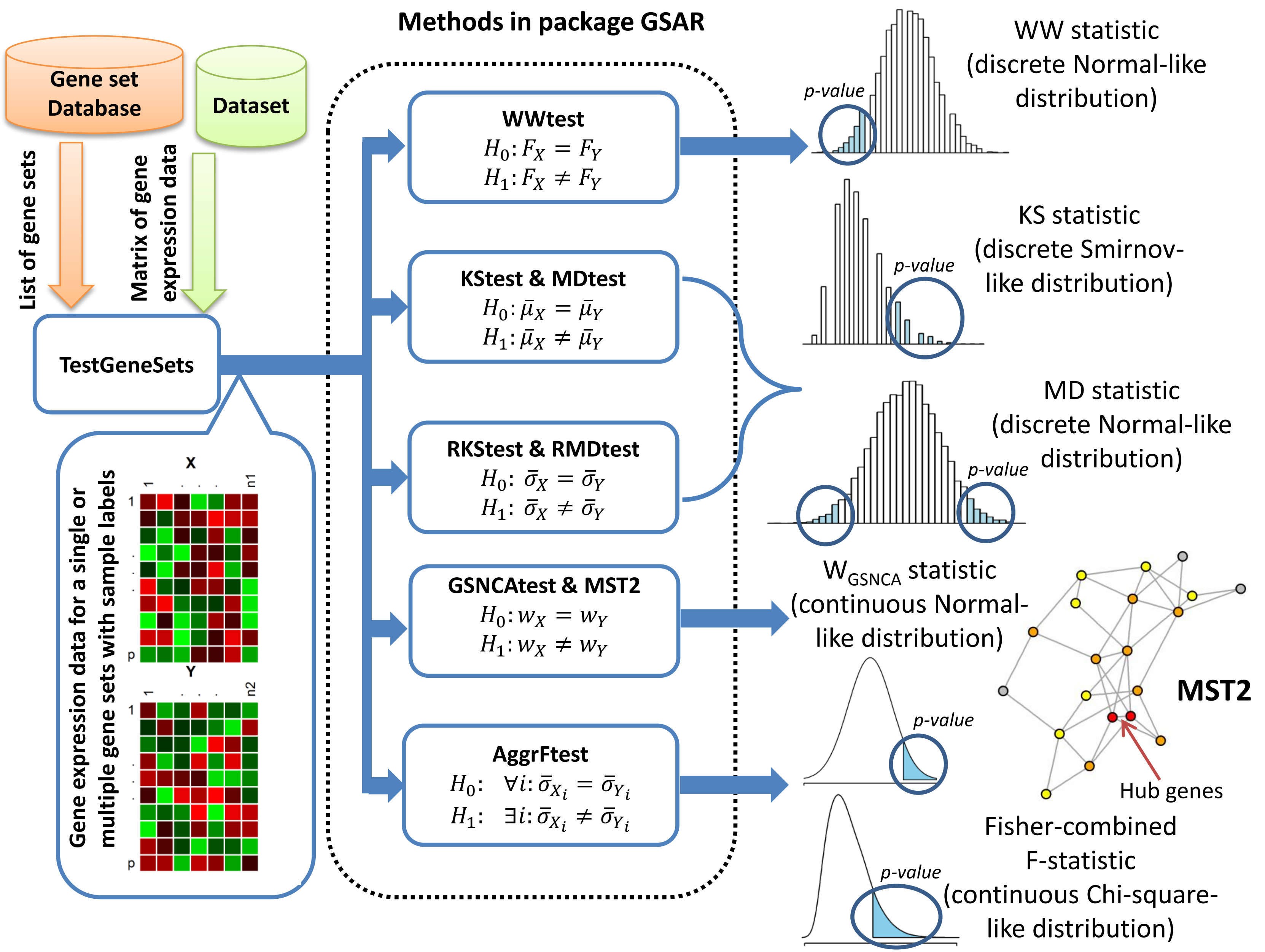
Methods in package GSAR
Gene set Database
Dataset
List of gene sets
Matrix of gene expression data
TestGeneSets
Gene expression data for a single or multiple gene sets with sample labels
X
Y
WWtest
$H_0: F_X = F_Y$
$H_1: F_X \neq F_Y$
KStest & MDtest
$H_0: \bar{\mu}_X = \bar{\mu}_Y$
$H_1: \bar{\mu}_X \neq \bar{\mu}_Y$
RKStest & RMDtest
$H_0: \bar{\sigma}_X = \bar{\sigma}_Y$
$H_1: \bar{\sigma}_X \neq \bar{\sigma}_Y$
GSNCAtest & MST2
$H_0: w_X = w_Y$
$H_1: w_X \neq w_Y$
AggrFtest
$H_0: \forall i: \bar{\sigma}_{X_i} = \bar{\sigma}_{Y_i}$
$H_1: \exists i: \bar{\sigma}_{X_i} \neq \bar{\sigma}_{Y_i}$
p-value
WW statistic (discrete Normal-like distribution)
KS statistic (discrete Smirnov-like distribution)
MD statistic (discrete Normal-like distribution)
W_{GSNCA} statistic (continuous Normal-like distribution)
MST2
Hub genes
Fisher-combined F-statistic (continuous Chi-square-like distribution)
